# Uncovering the Unusual Inhibition Mechanism of a Trypanosome Alternative Oxidase Inhibitor Displaying Broad-Spectrum Activity against African Animal Trypanosomes

**DOI:** 10.1021/acs.jmedchem.5c00631

**Published:** 2025-06-04

**Authors:** Godwin U. Ebiloma, Emmanuel O. Balogun, Natsumi Arai, Momoka Otani, Cecilia Baldassarri, Amani Alhejely, Eduardo Cueto-Díaz, Harry P. De Koning, Christophe Dardonville, Tomoo Shiba

**Affiliations:** † School of Science, Engineering & Environment, 7046 University of Salford, Manchester M5 4NT, United Kingdom; ‡ Graduate School of Science and Technology, Department of Applied Biology, Kyoto Institute of Technology, Kyoto 606-8585, Japan; § Department of Biochemistry, Ahmadu Bello University, Zaria 2222, Nigeria; ∥ Department of Biomedical Chemistry, Graduate School of Medicine, The University of Tokyo, Tokyo 113-0033, Japan; ⊥ Center for Discovery and Innovation in Parasitic Diseases, Skaggs School of Pharmacy and Pharmaceutical Sciences, University of California San Diego, 9500 Gilman Drive, La Jolla, California 92093, United States; # Medicinal Chemistry Unit, School of Pharmacy, Chemistry Interdisciplinary Project (ChIP), 18959 University of Camerino, Via Madonna delle Carceri, 62032 Camerino, Italy; ∇ Biology Department, Darb University College, 123285 Jazan University, Jazan 82817-2820, Saudi Arabia; ○ Instituto de Química Médica, IQM−CSIC, Juan de la Cierva 3, E−28006 Madrid, Spain; ◆ School of Infection and Immunity, College of Medical, Veterinary and Life Sciences, 3526 University of Glasgow, Glasgow G43 2DX, United Kingdom

## Abstract

The glucose-dependent respiration of bloodstream forms of the parasite *Trypanosoma brucei* depends on an unusual and essential mitochondrial electron-transport system, consisting of glycerol-3-phosphate dehydrogenase and the trypanosome alternative oxidase (TAO). We report here the discovery of an allosteric inhibitor of TAO that displays highly potent activity (EC_50_ values in the range 1–20 nM) against the important veterinary pathogens *T. b. brucei*, *Trypanosoma evansi*, *Trypanosoma equiperdum*, and *Trypanosoma congolense*, i.e., >5-fold greater potency than the standard drugs. The methylene-linked 2-methyl-4-hydroxybenzoate 2-pyridinyldiphenylphosphonium derivative (**1**) was the best inhibitor of recombinant TAO (IC_50_ = 1.3 nM) via a noncompetitive/allosteric mechanism (*K*_i_ = 3.46 nM). Remarkably, X-ray crystallography showed that **1** was bound to a site of TAO ∼25 Å from the catalytic pocket. Although **1** demonstrated good safety toward mammalian cells *in vitro* (selectivity index >2300), it did not fully clear parasitemia in experimental animals, attributable to a high hepatic clearance.

## Graphical abstract

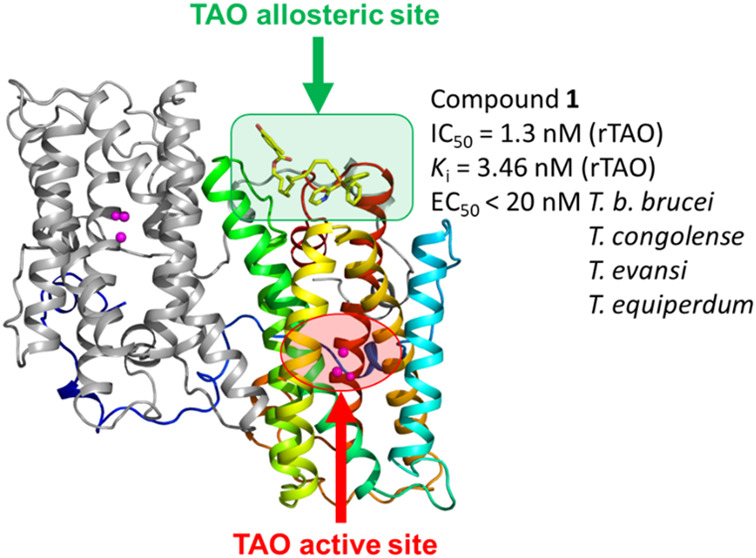

## Introduction

Trypanosomes are protozoan parasites of humans and livestock which, depending on the species, cause human African trypanosomiasis (HAT or sleeping sickness) and/or Animal African Trypanosomiasis (AAT or nagana).^1,2^ HAT and AAT are diseases with major clinical and economic impacts in many parts of sub-Saharan African countries; both can be fatal if not treated.^3,4^ Two subspecies of *Trypanosoma brucei* are responsible for the human form of the disease, namely *Trypanosoma brucei gambiense*, present in west and central Africa and accounting for over 98% of all reported cases of HAT, and *Trypanosoma brucei rhodesiense*, present in southern and eastern parts of Africa and responsible for less than 2% of the reported cases.^5–8^ There are two stages of HAT manifestation: hemolymphatic (early) stage with few specific symptoms, followed by a neurological stage where the parasites infect the central nervous system, which will then invariably lead to the death of the patient if untreated.^3^ However, for *T. b. gambiense* disease, nonsymptomatic carriers and apparent self-cure have been reported when progression to the cerebral stage did not occur.^9,10^ *T. b. rhodesiense* infection is characteristically acute, developing to the neurologic stage within a few weeks, and death could follow in months.^11^

AAT, on the other hand, is not limited to tropical regions of Africa, but is also endemic in South America and parts of Asia.^12^ It is caused by several trypanosome species, notably *T. b. brucei*, *Trypanosoma congolense*, *Trypanosoma vivax*, *Trypanosoma evansi*, *Trypanosoma equiperdum*, *Trypanosoma godfreyi*, and *Trypanosoma simiae*.^13,14^ *T. equiperdum* and *T. evansi* evolved from a common ancestor and are morphologically indistinguishable parasites, though they acquired key biological differences, including mode of transmission, pathogenicity, clinical symptoms, distribution, and host range.^15^ While human trypanosomiasis and most animal trypanosomiasis in Africa are transmitted by tsetse flies (Diptera: Glossinidae), *T. vivax*, *T. equiperdum*, and *T. evansi* do not depend on tsetse vectors and consequently have a wide range of distribution throughout the world. *T. vivax* and *T. evansi* are transmitted by biting insects, particularly *Stomoxys* and tabanid flies, whereas *T. equiperdum* spreads through copulation.

Animal trypanosomiasis constitutes a significant limitation for livestock farming and draft power and is known by various names, depending on the country, host, and infecting species. Surra is a wasting disease in equines, camels, and cattle caused by *T. evansi*, and results in great economic losses.^16,17^ Dourine is a notifiable venereal disease in horses and donkeys caused by *T. equiperdum*. While control of surra depends exclusively on chemotherapy, dourine is considered incurable, requiring the slaughter of infected animals.^18,19^ In Africa, animal trypanosomiasis of cattle is mostly known as nagana; the predominant etiological agents are *T. b. brucei*, *T. congolense*, *T. vivax*, and *T. b. rhodesiense* and control depends almost exclusively on treatment with either diminazene aceturate or isometamidium chloride.^4,20,21^

The disease can be treated and controlled through chemotherapy; however, nearly all the available treatment options have faced severe challenges, especially the emergence of resistant strains of trypanosomes but also their high toxicity, which prevents the use of increased dosages.^2,21^ For decades, treatment options for HAT have relied on these toxic drugs, including arsenicals for late-stage sleeping sickness, that typically require hospitalization of patients and long administration protocols (Chart 1, A). Despite the above challenges, significant gains have been made toward eliminating the disease, and these efforts have also contributed to the recent development of the newer generations of drugs, fexinidazole and acoziborole (Chart 1, B), both of which are orally administered drugs. Fexinidazole was successfully registered for treating both stages of gambiense HAT^21–23^ and is now also recommended for rhodesiense sleeping sickness. Although the introduction of a single oral treatment for sleeping sickness, regardless of species or disease stage, constitutes a true milestone, some challenges remain. For instance, fexinidazole is too toxic to be used for severely ill patients or for young children and must be administered by trained health professionals.^23,24^ Moreover, no new drugs against animal trypanosomiasis have been introduced for decades.^2,25^

**Chart 1:**
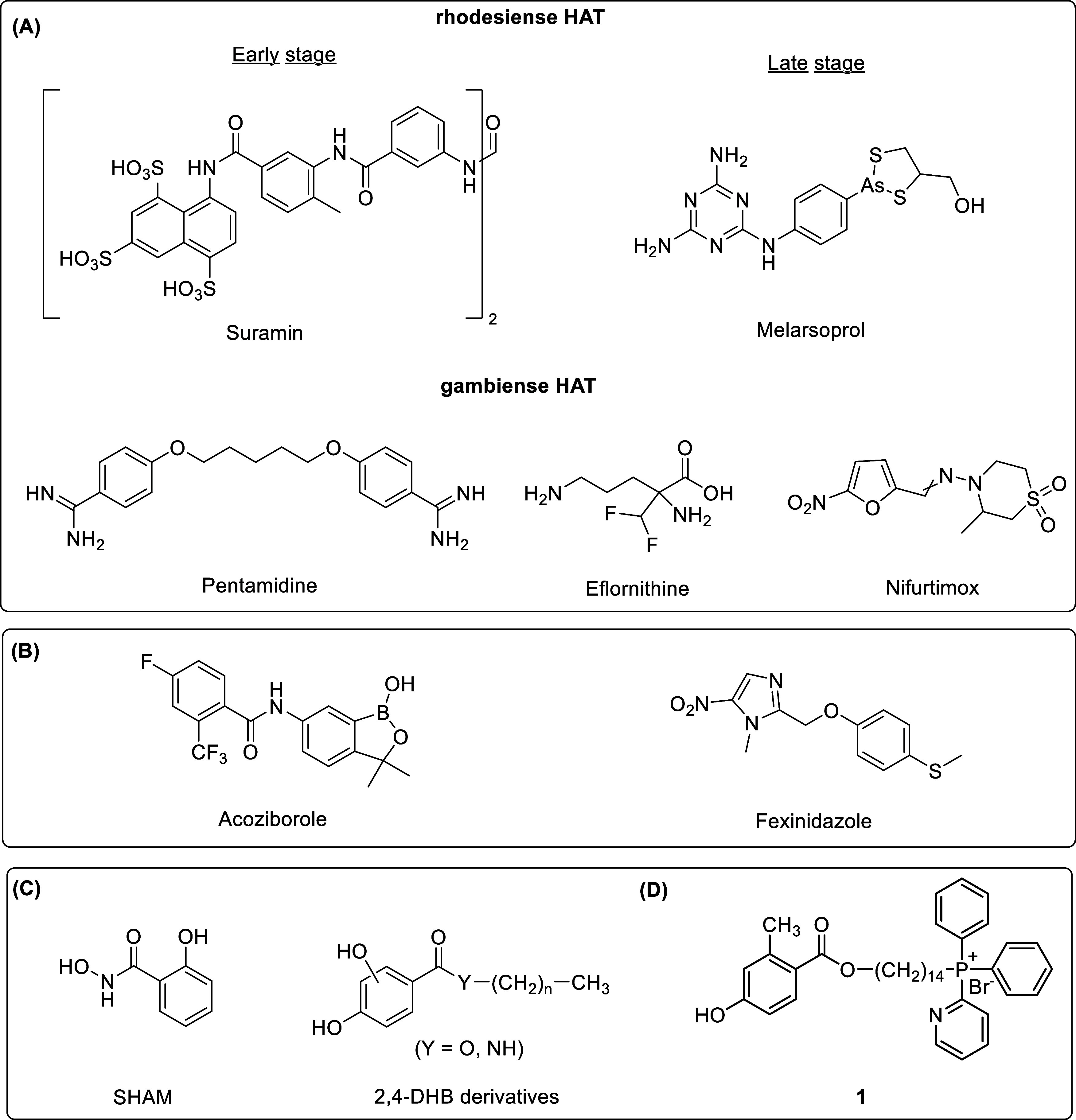
(A) Historical Drugs Used for the Treatment of Gambiense and Rhodesiense HAT. (B) New Orally Active Drug Registered for Treating Both Stages of Gambiense HAT. (C) Structurally Related Early (Micromolar) TAO Inhibitors: Salicylhydroxamic Acid (SHAM) and 2,4-Dihydroxybenzoate (2,4-DHB) Derivatives. (D) Mitochondrion-Targeted (Nanomolar) Allosteric TAO Inhibitor, Compound **1**

Among the most promising targets for the development of new trypanocides are essential mitochondrial enzymes that are part of the parasite’s glycolysis-dependent respiratory pathway.^26–29^ Among the various validated mitochondrial targets in trypanosomes (e.g., fatty acid biosynthesis, tRNA import, kinetoplast DNA and topoisomerases, etc.),^30^ the mitochondrial respiration of the parasite is a particularly attractive target as it is dramatically different from the mammalian host’s.^31^ In effect, throughout their life cycle, the energy metabolism of trypanosomes adapts to the available nutrients in their environment.^32^ Consequently, the procyclic forms of the parasite, located in the midgut of the tsetse fly vector (a glucose-poor environment), have a fully functional respiratory chain and synthesize ATP via oxidative phosphorylation in the mitochondrion. In contrast, the long, slender bloodstream trypomastigotes of *T. brucei*, present in the mammalian bloodstream, depend entirely on glycolysis for their energy production, and lack the enzymes of oxidative phosphorylation, the tricarboxylic acid cycle, and the canonical electron transport systems.^33^

Clarkson et al. first demonstrated that the respiratory pathway of the *T. brucei* bloodstream trypomastigotes is solely dependent on a “plant-like alternative oxidase” now known as the trypanosome alternative oxidase (TAO), which is located in the inner mitochondrial membrane of the parasite.^34^ Because TAO has no counterpart in any mammalian host, and because it is essential for the viability of bloodstream trypanosomes, TAO is considered an excellent target for chemotherapy.^35–38^

Very simple chemical structures built on the 2,4-salicylhydroxamate (SHAM) and dihydroxybenzoate (2,4-DHB) scaffolds (Chart 1, C) were shown to inhibit TAO.^39–42^ However, the trypanocidal activity of these compounds proved rather disappointing due to the inhibitors’ inability to sufficiently accumulate across the cell and mitochondrial membranes to reach their target. We reported recently that the coupling of mitochondrion-targeting lipophilic cations to TAO inhibitors significantly improved mitochondrial targeting and trypanocidal activity while retaining target protein potency.^26–28,31,43,44^

In a systematic effort to improve the trypanocidal activity of the 2,4-DHB scaffold and study for the first time their mode of TAO inhibition, we here report the SAR studies and discovery of compound **1** (Chart 1), a new low-nanomolar range TAO inhibitor with broad activity against wild-type and drug-resistant African trypanosomes. This compound is the first TAO inhibitor featuring a 2-pyridinyldiphenylphosphonium (PDPP) cation. The in vitro mode of action of **1** against *T. brucei* was determined by direct inhibition and kinetic studies on recombinant, purified TAO (rTAO). The crystal structure of the TAO–**1** complex provided a molecular basis for the observed inhibition of TAO, revealing a surprising new allosteric mode of inhibition. A preliminary efficacy experiment in the STIB795 mouse model of *T. b. brucei* infection was performed to assess the in vivo potential of hit compound **1**.

## Results

### Chemistry

Compounds **1**–**12** were prepared as described in Scheme 1. The reaction of 1,14-dibromotetradecane^45^ with commercially available 4-hydroxy-2-methylbenzoic acid, 2,4-dihydroxy-6-methylbenzoic acid, and 2,4-dihydroxybenzoic acid, or 3,5-dichloro-2,4-dihydroxy-6-methylbenzoic acid,^26^ yielded intermediate compounds **13**–**16**, respectively (Scheme 1).^27^ The reaction of **13** and **14** with diphenyl­(2-pyridinyl)­phosphine in acetonitrile at 80 °C for 6 days produced **1** and **2** with approximately 40% yield and >95% purity after silica chromatography. The synthesis of **3**, **5**, and **11**, following the synthetic route shown in Scheme 1 (and their in vitro antileishmanial activity) has been reported before.^46^ Their IC_50_ against rTAO is reported here for the first time (Figure 1). The synthesis (shown in Scheme 1) and in vitro activity of **4**, **6**, **8**–**10**, and **12** against TAO and *T. brucei* have been reported earlier.^26–28^ Chlorinated compound **7**, which is reported here for the first time, was synthesized by reaction of **16** with Ph_3_P in refluxing acetonitrile (62% yield).

**Figure 1:**
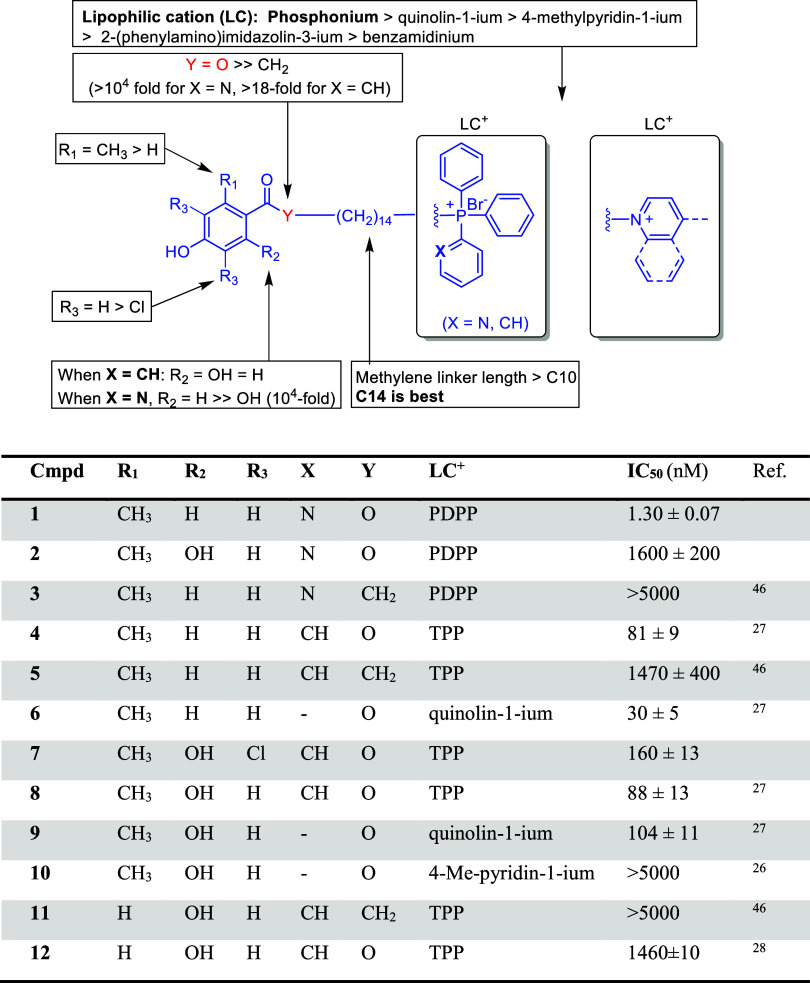
IC_50_ values against rTAO and SAR of triarylphosphonium TAO inhibitors (PDPP = 2-pyridinyldiphenylphosphonium, TPP = triphenylphosphonium). Compound **1** is the most potent TAO inhibitor among all the phosphonium series we have reported so far.^26–28,43,44^ Reference compound: ascofuranone, IC_50_ = 2 nM (Table 1). All IC_50_ values are the average and SEM of 4 independent determinations.

**Scheme 1:**
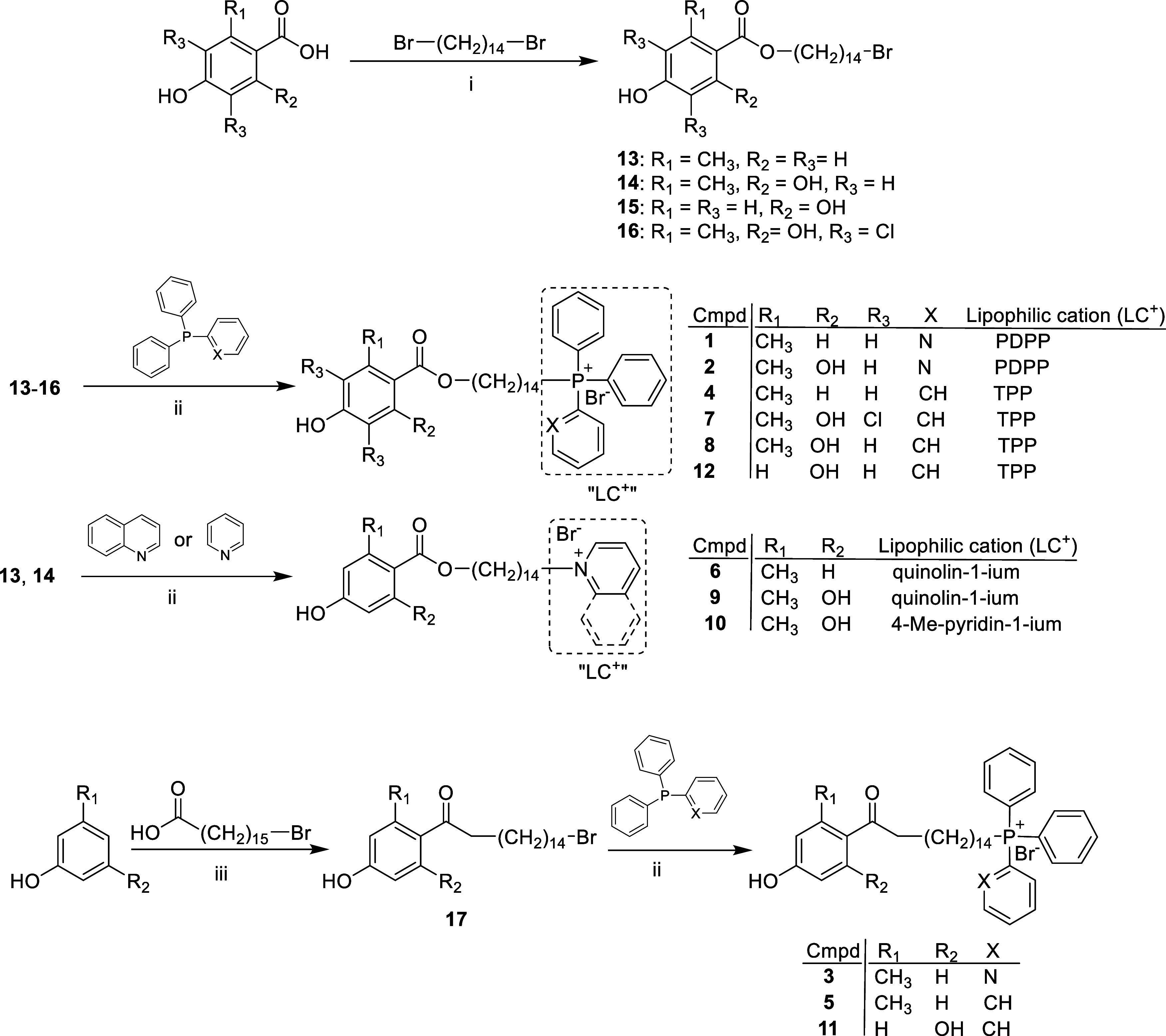
Synthesis of Compounds **1**–**12** [Fn s1fn1]

^a^
Reagents and conditions: (i) NaHCO_3_, 65 °C, CH_3_CN, 9 days (39%); (ii) CH_3_CN, 80 °C, 6 days (39–62%); (iii) BF_3_–Et_2_O, 110 °C.

### SAR Studies of 2,4-DHB Inhibitors with rTAO

The compounds were tested as inhibitors of the ubiquinol oxidase activity of purified recombinant TAO (rTAO) by noting the change in absorbance of ubiquinol-1 at 278 nm, using SHAM and ascofuranone, two known TAO inhibitors, as positive controls. The negative control, DMSO, did not affect rTAO activity. Earlier SAR studies with 2,4-DHB inhibitors of TAO showed that a minimum length of the methylene linker (>C10) and a certain level of lipophilicity in the tail region of the molecule was required for potent inhibition (Figure 1).^31^ In agreement with these findings, we showed that a linker length of 14 carbons was preferred for TAO inhibition by dihydroxybenzoate triphenylphosphonium (TPP) derivatives.^27,28,44^ The nature of the lipophilic cation (LC) is also determinant for TAO inhibition; the TPP group being generally superior to the quinolin-1-ium > 4-methylpyridin-1-ium > 2-(phenylamino)­imidazolin-3-ium > benzamidinium (Figure 1: compare **4** vs **6**; **8** vs **9** and **10**).^26,27,31,43,44^

In the present work, compound **1** displayed low nanomolar inhibition against rTAO (1.3 ± 0.07 nM), which is in the same range as ascofuranone (2.0 ± 0.4 nM)—a known antibiotic inhibitor of TAO^47^—and it was even better than SHAM (>1900 times better). In contrast, compound **5** was a 1000-fold weaker inhibitor (1.47 μM), whereas compounds **3** and **11** did not inhibit TAO at the highest concentration tested (5 μM).

We observed that changing the ester linker (Y = O) to a more metabolically stable keto bond (Y = CH_2_) was detrimental to TAO inhibition (Figure 1: compare **1** vs **3**; **4** vs **5**; **11** vs **12**). Of note, this inhibitory activity loss was more pronounced for X = N (>10^4^-fold) than for X = CH (18-fold). Remarkably, the introduction of a pyridine ring in the lipophilic cation to form a 2-pyridinyldiphenylphosphonium (PDPP) cation (**1**: X= N, IC_50_ = 1.3 nM) improved TAO inhibition 62-fold with respect to the TPP analog **4** (X = CH, IC_50_ = 81 nM), yielding one of the most potent (nanomolar) TAO inhibitors reported to date and the best among all the phosphonium series we have reported so far. Surprisingly, this effect seemed to be restricted to the 2-methyl-4-hydroxybenzoate scaffold (**1**, R_1_ = CH_3_, R_2_ = H, Y = O) as shown by the >1200-fold higher IC_50_ value of the 2,4-dihydroxy-6-methylbenzoate derivative **2** (R_1_ = CH_3_, R_2_ = OH, Y = O; IC_50_ = 1.6 μM) and keto analog **3** (R_1_ = CH_3_, R_2_ = H, Y = CH_2_; IC_50_ > 5 μM). In contrast, the detrimental effect of adding a 2-OH group was not observed in the TPP series where compounds **4** (R_2_ = H) and **8** (R_2_ = OH) showed a similar inhibitory potency (≈80 nM).^27^ In fact, the 2,4-dihydroxy-6-methylbenzoate TPP derivative **8** (IC_50_ = 88 nM) was 26 times more potent than its PDPP analog **2** (IC_50_ = 1.6 μM).

The introduction of two chlorine atoms in the aromatic ring of **8** (R_3_ = H, IC_50_ = 88 nM) was somewhat counterproductive and yielded the 2-fold weaker inhibitor **7** (R_3_ = Cl, IC_50_ = 160 nM). Hence, according to the SAR results reported earlier for monochlorinated 4-methyl-pyridin-1-ium compounds^26^ and ascofuranone derivatives,^31^ a single chlorine atom in R_3_ next to R_2_ may be beneficial, although this hypothesis would need to be tested.

Altogether, these intriguing SAR results show that low nanomolar inhibition of TAO is dependent on subtle changes in the structure of the 2,4-DHB scaffold and the methylene-linked phosphonium cation, suggesting that the TPP and PDPP derivatives have distinct modes of binding to TAO. Hence, we decided to study in depth the in vitro potential of hit compound **1** against trypanosomes and to characterize its inhibition mode by kinetic and crystallographic studies. Overall, the structure of the TAO*–* **1** complex showed an important contribution from the pyridine nitrogen of the PDPP derivative to binding in the allosteric site of TAO.

## Biology

### Effect of **1** against Wild-Type and Drug-Resistant Strains of Trypomastigotes of Animal-Infective Trypanosomes

Compound **1**, the most potent TAO inhibitor of the series, is active in the single-digit nanomolar range against trypomastigotes of *T. b. brucei*, *T. evansi*, and *T. equiperdum* (Table 1), including drug-resistant strains (Table 2). There was no significant difference (*p* < 0.05) in the EC_50_ of the compound against the three species, although **1** was slightly more active against *T. evansi* than *T. b. brucei* (0.0012 and 0.0031 μM, respectively). These results also show that **1** is slightly more active against these trypanosome species, all of the *Trypanozoon* subgenus, than the trypanocidal drug pentamidine (0.0042 μM) and much more active than the standard veterinary drug diminazene aceturate (0.065 μM). However, activity against *T. congolense* bloodstream forms (subgenus *Nannomonas*) was lower than against *T. brucei*, with an EC_50_ of 0.041 μM—in line with similar differences observed previously with TAO inhibitors.^27,28^ Nevertheless, **1** was over 6-fold more potent against *T. congolense* than the standard treatment diminazene (Table 1) and considering the widespread resistance to diminazene in *T. congolense*,^2,48^ this is nonetheless an important observation.

**Table 1:** EC_50_ Values (μM) against Wild-Type Strains of Trypomastigotes of Animal Infective Trypanosomes, Cytotoxicity against Human Cells (CC_50_, μM), and Inhibition of Recombinant TAO (IC_50_, μM)

		*T. b. brucei* WT (+5 mM glycerol)^b^		T. evansi WT	T. equiperdum WT	T. congolense WT	HEK^e^		rTAO^f^
compd	*T. b. brucei* WT^a^ (no glycerol)	EC_50_	RF^c^	EC_50_	EC_50_	EC_50_	EC_50_	SI^d^	IC_50_
**1** ^g^	0.0031 ± 0.0006	0.0014 ± 0.0005	0.45	0.0012 ± 0.0001	0.0022 ± 0.0002	0.041 ± 0.007	7.49 ± 0.10	2385	0.0013 ± 0.00007
Pent.^h^	0.0042 ± 0.0008	0.0062 ± 0.0017	1.49	0.0051 ± 0.0021	0.0081 ± 0.0011				
Dim.^i^	0.065 ± 0.021	0.081 ± 0.013	1.15	0.0514 ± 0.012	0.0332 ± 0.010	0.252 ± 0.018			
SHAM^j^	65.02 ± 0.36	17.76 ± 3.57	0.27						5.93 ± 0.13
PAO^k^							0.29 ± 0.02		
AF^l^									0.0020 ± 0.0004

a Trypomastigotes of *T. b. brucei* s427 (*n* ≥ 4).

b *T. b. brucei* incubated in the presence of 5 mM glycerol.

c Resistance factor relative to *T. b. brucei* WT without glycerol: RF = EC_50_ (in the presence of glycerol)/EC_50_ (without glycerol).

d Selectivity index (SI) = CC_50_ of HEK/EC_50_ of *T. b. b* WT (no glycerol).

e Cytotoxicity on Human Embryonic Kidney (HEK) 293-T cells (*n* = 3).

f Recombinant TAO enzyme.

g The antileishmanial activity of **1** has been reported previously.^46^

h Pentamidine isethionate.

i Diminazene aceturate.

j Salicylhydroxamic acid.

k Phenylarsine oxide.

l Ascofuranone. *, *P* < 0.05, **, *P* < 0.01 relative to control without glycerol.

**Table 2:** EC_50_ Values (μM) of **1** against Drug-Resistant *T. brucei* Trypomastigotes

	*T. brucei* B48^a^		*aqp1-3* null^b^		SUR10^c^	
compd	EC_50_	RF^d^	EC_50_	RF^d^	EC_50_	RF^d^
**1**	0.0025 ± 0.0001	0.8	0.0012 ± 0.00013	0.38	0.0036 ± 0.0009	1.14
Pent.^e^	1.02 ± 0.20	243	0.0514 ± 0.0118	12.1	nt	
Dim.^f^	1.22 ± 0.02	4.6	nt		nt	
Suramin	nt		nt		0.19 ± 0.02	21.1
SHAM^g^	nt		6.88 ± 0.11	0.11	nt	

a Trypomastigotes of *T. b. brucei* strain resistant to pentamidine, diminazene, and melaminophenyl arsenicals (*n* ≥ 4).

b Bloodstream form (BSF) *T. brucei* Aquaporin1–3 triple knockout.

c BSF of *T. b. brucei* rendered resistant to suramin by in vitro exposure.

d Resistance factor relative to *T. b. brucei* wild type (see Table 1).

e Pentamidine.

f Diminazene aceturate.

g Salicylhydroxamate. The experiments with the various *T. brucei* clones reported in Tables 1 and 2 were performed in parallel and have the same controls and experimental conditions, where necessary for side-by-side comparisons. *, *P* < 0.05; **, *P* < 0.01; ***, *P* < 0.001 relative to WT control in unpaired *t* test; nt, not tested.

Compound **1** cytotoxicity against the human cell line HEK was drastically lower compared to that of *T. b. brucei* (7.49 and 0.0031 μM, respectively), resulting in a selectivity index (SI) of 2385.

Co-incubation with glycerol, which inhibits the *T. brucei* anaerobic ATP production pathway,^49^ significantly (*P* < 0.05) increased the trypanocidal activities of **1** and SHAM, whereas 5 mM glycerol did not potentiate the control drugs pentamidine isethionate and diminazene aceturate (Table 1). This result is consistent with the aerobic glycolytic pathway being a target of SHAM and other compounds reported to be inhibitors of TAO^26,27,43,44,47^ whereas diamidine trypanocides accumulate in the kinetoplast.^50,51^

Compound **1** was tested against drug-resistant strains of *T. brucei*. The results showed that there was very little difference in activity between WT and B48 cell lines, with resistance factors (RF) consistently close to 1 (Table 2), indicating that the known drug transporters TbAT1/P2 and HAPT1/TbAQP2, absent from the B48 clone,^52,53^ are not involved in their uptake. This signifies that there is no cross-resistance of **1** with the current diamidine and arsenical trypanocides. In addition, there appears to be no cross-resistance with the important trypanocidal drug suramin, as the EC_50_ for the suramin-resistant cell line SUR10 was not drastically different from that for s427 (Table 2). Suramin resistance is of particular concern in the treatment of surra in camels.^54^

It is known that the inhibition of TAO forces trypanosomes to resort to anaerobic glycolysis to generate ATP for survival and, in the process, produce glycerol in large amounts via the reverse activity of their glycerol kinase.^55^ Hence, in the absence of the aquaglyceroporin glycerol–water channels, trypanosomes are not able to efficiently dispose of that large amount of glycerol from their cell^56^ and consequently become more susceptible to the TAO inhibitors. Accordingly, a *T. brucei* cell line from which all the aquaporins were knocked out (*aqp1-3* null) was used in this research.

The result shows that **1** was significantly more effective (*P* < 0.05) against the *aqp1-3* null (Table 2), resulting in a resistance factor of 0.38, indicating increased sensitivity to the test compound. The observed effect of compound **1** on the *aqp1-3* null line may be explained as a consequential shift in trypanosomes’ energy metabolism in the presence of **1**. The TAO inhibitor SHAM, used as positive control, was also significantly more effective (*p* < 0.001) against the *aqp1-3* null line (Table 2).^56^

### Compound **1** Affects Trypanosomes Irreversibly after a Limited Exposure Time

The results in Figure 2, which show the growth curve of *T. brucei* wild type s427 grown in the continuous presence or after a limited exposure (24 h) to **1**, indicate that the growth pattern of cells with long-time exposure was similar to that of cells with limited exposure to **1**. The compound seemed to rapidly induce an apparent growth inhibition, after approximately 4h, followed by a steep decline in population from 30 h. In the presence of 5 × EC_50_, the trypanocidal phase happened somewhat earlier, and steeper, than at 2 × EC_50_. There was no difference in the growth pattern between the continuous presence vs the 24 h exposure, which indicates that irreversible damage to the cells is already inflicted during the initial growth inhibition. Thus, the effects of **1** on trypanosomes are irreversible before population decline begins.

**Figure 2:**
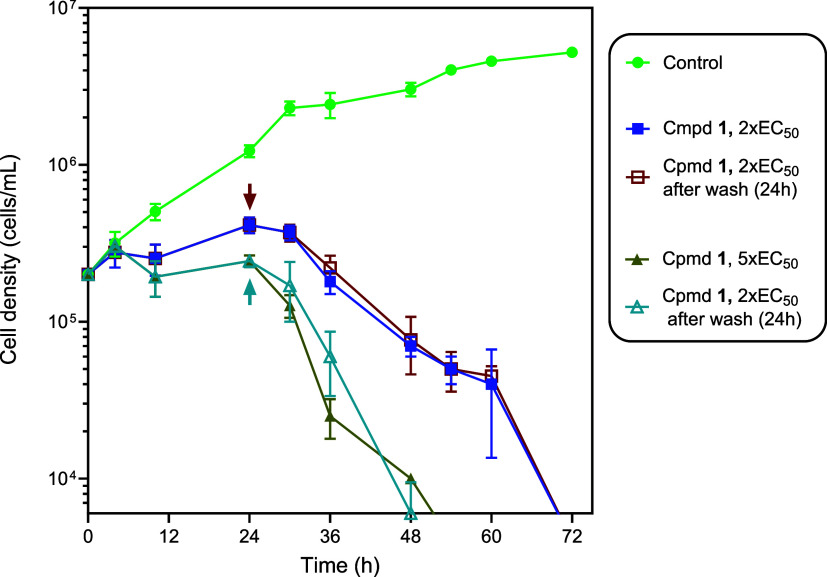
Growth curve of *T. brucei* wild type s427 grown in either the continuous presence of **1** at indicated concentrations (filled symbols) or with the drug washed out with fresh media after 24 h (open symbols; the arrows indicate the time when compound **1** was withdrawn), or untreated (no drug control). The results presented are those of three biological repeats. Error bars represent SD of the biological replicates.

### Rapid Onset of Growth Inhibition is Associated with G1 Growth Arrest

To assess the cause of the apparent 24-h growth arrest visualized in Figure 2, we assessed the cellular DNA content by flow cytometry of fixed trypanosomes stained with the nucleic acid dye propidium iodide after RNase treatment. The percentage of cells containing a DNA content consistent with a single diploid set of chromosomes (G1 phase) increased immediately (first time point was 1 h) in compound **1** treated cells, while at the same time the number of cells with double the DNA content (G2 phase) or intermediate DNA content (S phase) rapidly decreased (Figure 3A). This indicates an inability to initiate new synthesis of nuclear DNA, while cells that already past that checkpoint were able to complete cell division. To verify our conclusion, we next stained cells exposed to 2 × EC_50_ “compound **1**” with 4,6-diamidino-2-phenylindole (DAPI) for fluorescence microscopy, visualizing both the nucleus and the kinetoplast with the mitochondrial DNA. In *T. brucei*, the first phase of the cell cycle is the replication of the kinetoplast, before the subsequent duplication of the nucleus.^57^ Thus, at the start of the experiment (*t* = 0 h) an equal percentage of cells in the “compound **1**” and “untreated control” cultures contained a single nucleus and kinetoplast (1N1K) or were in the initial stages of cell division with 2 kinetoplasts (1N2K) (Figure 3B). For the control, the percentage in 1N1K remained stable, whereas the exposure to **1** resulted in a progressive increase in 1N1K cells until they consisted of 100% of cells observed by 24 h. These observations strengthen our conclusion that treatment with **1** stops the initiation of a new cell division cycle, and specifically the replication of the kinetoplast that must precede nuclear DNA synthesis in trypanosomes. This leads to growth arrest of the cells in G1 phase (Figure 3A) and is followed by rapid population decline (cell death) after 24 h (Figure 2).

**Figure 3:**
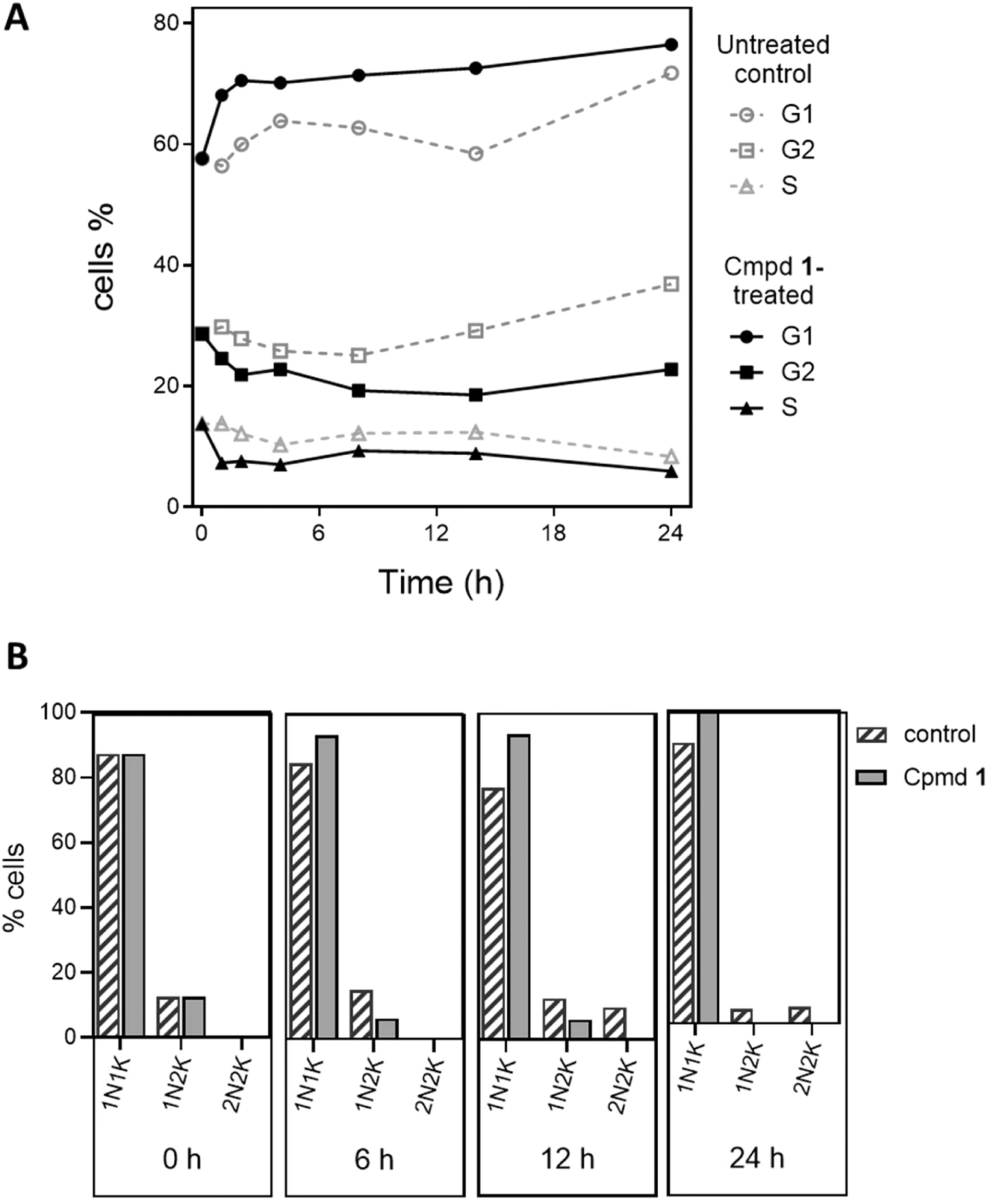
Assessment of DNA configuration. (A) Percentage of cells at various cell division stages in populations treated or not treated with 2 × EC_50_ over a 24-h period. The percentages are the average of three independent determinations and SEM, obtained using flow cytometry after cell permeabilization and staining with propidium iodide. (B) DNA content of cells treated over a 24 h period with compound 1 at 2 × EC_50_ as determined by fluorescence microscopy following DAPI staining. N, nuclear DNA; K, kinetoplast DNA. The percentages are the average of three independent determinations and SEM.

### Determination of TAO Kinetics in the Presence of Inhibitor **1**

#### Compound **1** is a Noncompetitive Nanomolar Range Inhibitor

We have previously reported the production of a more active “physiological” recombinant TAO enzyme that lacks the mitochondrial targeting sequence (ΔMTS rTAO) using a Small Ubiquitin-related Modifier (SUMO) expression system to optimize the production of rTAO.^27^ This physiologically relevant rTAO enzyme was used throughout this work and in this experiment to study the inhibitory activity of **1**.

To determine the type of inhibition with purified TAO, the kinetic assay was carried out in the presence of varying concentrations of **1** and substrate concentrations (Ubiquinol; Q_1_H_2_) (0–600 μM), with 250 ng TAO in a 1 mL (0.5 M Tris-HCl buffer, pH 7.3) reaction mix. The results were used to construct the Michaelis–Menten plot, which was then transformed to Lineweaver–Burk plots (Figure 4A). The *K*_m_ and *V*_max_ values for the uninhibited reaction were obtained as 189.6 ± 8.8 μM and 71.4 ± 0.003 μmol/min/mg, respectively. While the apparent *K*_m_ remained the same in the presence of different concentrations of compound **1**, as in its absence, *V*_max_ was progressively reduced in the presence of 5, 10, and 15 nM of compound **1** to 34.7, 21.4, and 13.0 μmol/min/mg, respectively (Figure 4A). The value of the inhibition constant, *K*_i_ was obtained as 3.45 nM from the Dixon plot (slopes of the lines in the reciprocal plots vs inhibitor concentrations) of the Lineweaver–Burk plot (Figure 4B). The test compound revealed a noncompetitive type of inhibition.

**Figure 4:**
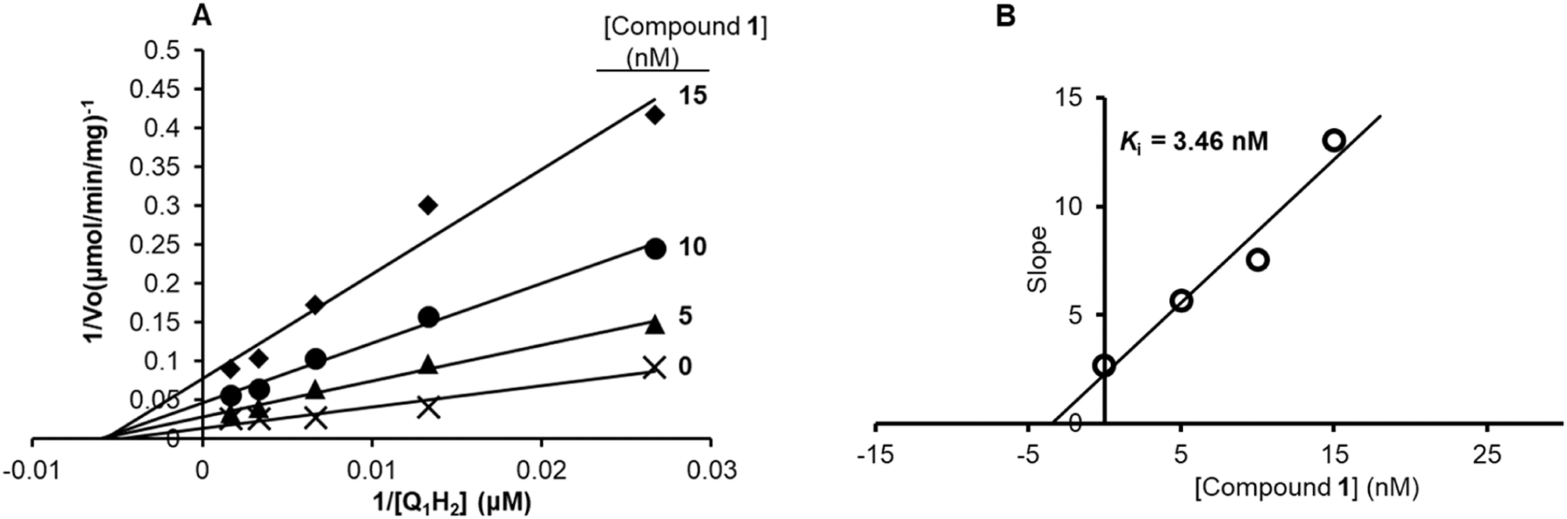
Inhibition mechanism and potency of compound **1** against TAO. (A) Lineweaver–Burk plots for TAO in the absence and presence of 5, 10, and 15 nM of the inhibitor. The curve revealed a noncompetitive inhibition pattern for **1** against TAO. (B) Dixon plot showing the *K*_i_ of **1** on TAO. The slopes of the Lineweaver–Burk primary plot were plotted against the varying concentrations of **1**. *K*_i_ = −(intercept over the *X*-axis) of the Dixon secondary plot.

### Crystal Structure of TAO–Compound **1** Complex: Insights into the Mode of Inhibition

#### Compound **1** Occupies an Allosteric Binding Site Distant from the Active Site

Via structure–activity relationship studies, we have established that the methylene-linked 2-methyl-4-hydroxybenzoate PDPP derivative **1** is, thus far, the most potent inhibitor of the alternative oxidase within that series.^27,28,44^ Herein, we investigated the inhibitory mechanism of **1** and established that, unlike ascofuranone, which is a competitive inhibitor of TAO,^58,59^ **1** has a noncompetitive inhibition mode on TAO as clearly shown by the Lineweaver–Burk plot (Figure 4A).

To obtain a better perspective of this novel inhibition mode, it was therefore important to obtain the structure of TAO in complex with **1** (TAO–**1**) in order to compare with the structures of other TAO-inhibitor complexes (PDB IDs 3VV9, 3VVA, 5ZDP, 5GN5).^60–62^ Such information could potentially enhance the optimization of **1** as a new class of TAO inhibitor. Figure 5 depicts the X-ray crystal structure of TAO–**1** at 3.01 Å (PDB ID: 9M2A). The data collection and refinement statistics are presented in Table S1. The crystal structure revealed that each crystal lattice was formed by two dimers (chains A–D) of TAO related by a noncrystallographic 2-fold axis and **1** was bound only in chain A (Figure 5A).

**Figure 5:**
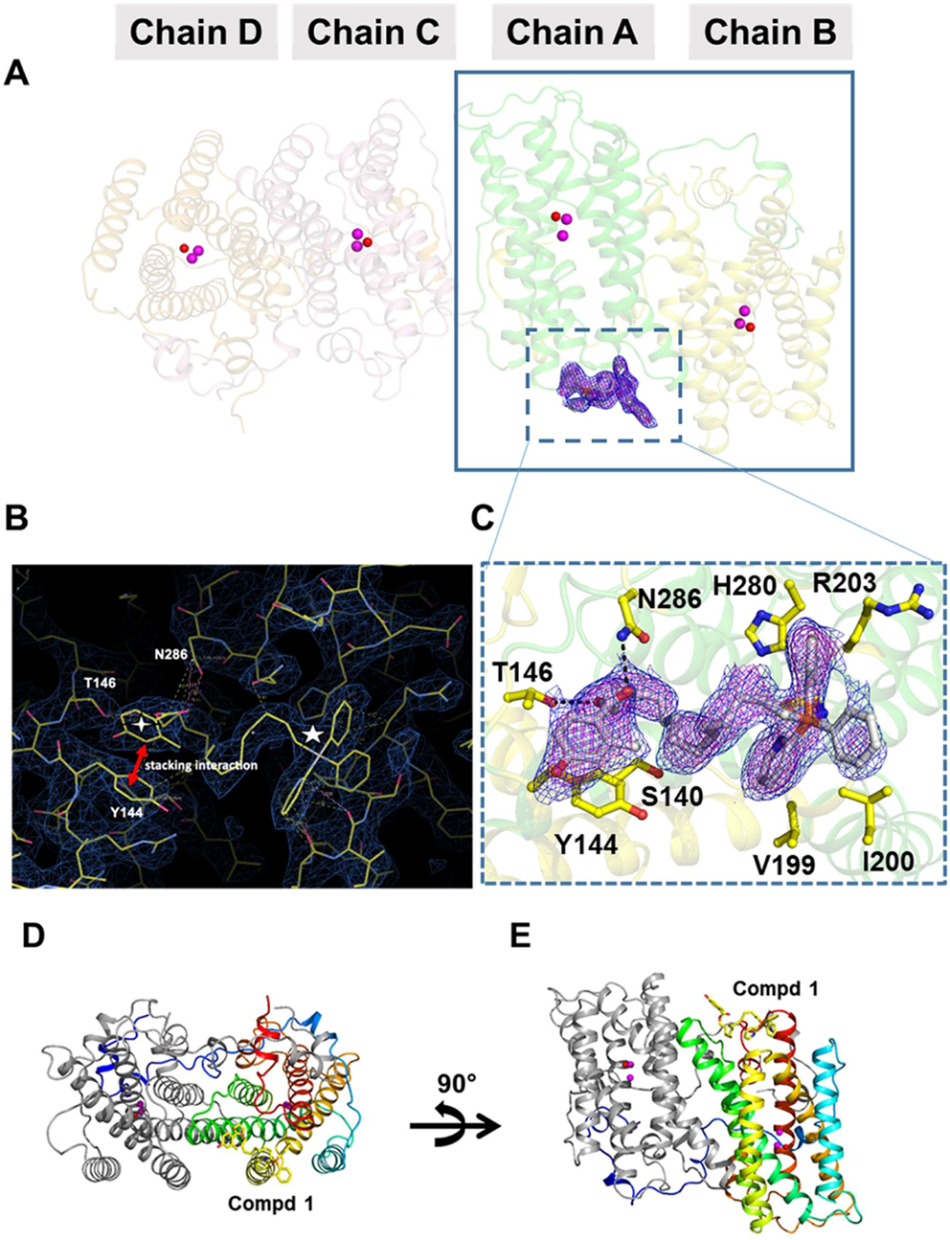
Structure of TAO–compound **1** complexes (PDB ID: 9M2A). (A) Complex structure of TAO–**1** in the crystal. Each crystal lattice was formed by two dimers of TAO—formed by chains A-B and C-D—related by a noncrystallographic 2-fold axis. Compound **1** was bound only to chain A. Compound **1** is shown in gray stick model with the 2Fo–Fc map contoured at 1.0 σ, showing the electron density for **1** bound far from the active site (the diiron center) of Chain A. Fe atoms and OH^–^ ions are respectively shown as purple and red spheres and present in the active site of all chains. (B) Stick model of X-ray crystallographic structure for rTAO–**1** complex. Compound **1** was bound to TAO by varied interactions with different amino acid residues at the binding site. The binding was established by hydrogen bonds and stacking interactions and hydrophobic interactions. The 4-hydroxy-2-methyl benzoic acid head is marked as 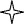, while the triphenylphosphonium tail as ☆, both linked by the methylene linker. The electron density map is shown contoured to 1.0 σ. (C) The amino acids binding compound **1**. The amino acid residues located within 4 Å of the **1** molecule are shown in stick models. The hydrogen bonds are shown in dash lines. The omit electron density map for molecule **1** is contoured 1.0 σ (blue) and 1.5 σ (purple), respectively. (D) Dimer structure of TAO–**1** complex. Chain A is shown in the rainbow cartoon from blue (N terminus) to red (C terminus), and chain B is gray. Compound **1** is shown in the yellow stick model. Fe atoms and OH^–^ ions are shown as purple and red spheres, respectively. Compound **1** is bound only to chain A, while the Fe atoms and OH^–^ ion are in both chains. (E) 90° orientation of chain D, revealing that **1** was bound far away (∼25 Å) from the active site of TAO. Fe atoms and OH^–^ ions present in the active site are shown as purple and red spheres, respectively.

It has been previously shown that the functional form of TAO exists as a homodimer.^60,62^ The electron density around compound **1** was very clear with good occupancy for all the chemical moieties of the inhibitor, from its 4-hydroxy-2-methyl benzoic acid head through the methylene linker to the 2-pyridyldiphenylphosphonium tail (Figure 5B). The molecular interactions utilized in maintaining **1** at the binding site included π-stacking interactions involving rings of the benzoic acid head and neighboring tyrosine (Y144) of TAO, extensive hydrogen bonds, including with T146 and N286, and electrostatic interactions (Figure 5B). Of note, strong multiple H-bonds between the pyridine N in the tail portion of **1** (i.e., PDPP cation) with backbone carboxyl groups of V199 and I200 of TAO significantly contributed to stabilizing the binding of **1** to TAO (Figure 5B). In the TPP analog **4**, the absence of this N atom is associated with a 62-fold drop in inhibitory potency and a shift toward competitive inhibition for the quinolin-1-ium (**6**) and TPP (**8**) analogs (Figure 1).^27^ Hence, it appears that the pyridinyl nitrogen is crucial for binding to the allosteric site of TAO. The amino acid residues that bind the inhibitor are R137, S140, Y144, T146, V199, I200, P202, R203, H280, N286 (Figure 5C). The only difference between compounds **1** (IC_50_ = 1.3 nM) and **2** (IC_50_ = 1600 nM) is the addition of a 2-OH group in the benzoic acid head in **2**. Thus, the unfavorable interaction of **2** with tyrosine Y144 of the binding site (due to OH–OH repulsion) may explain why **1** is a nanomolar range inhibitor whereas **2** is not (Figure 5B). The binding site for **1** is unique in that it is found far from the active site of TAO, at a distance of approximately 25 Å (Figure 5D and E). The active site of TAO is mapped by the presence of di-iron and a hydroxo ion (Figure 5A, D, and E). This unique (allosteric) binding site of compound **1** supports the inhibition kinetics data (Figure 4A) and further underpins our conclusion of compound **1** as a noncompetitive inhibitor of TAO. Further, **1** was bound at helices 1 and 4 of TAO (Figure 6D and E), which form the membrane anchor of TAO.^60^ Hence, the binding of **1** may interfere with both the membrane binding and functionality of TAO.

**Figure 6:**
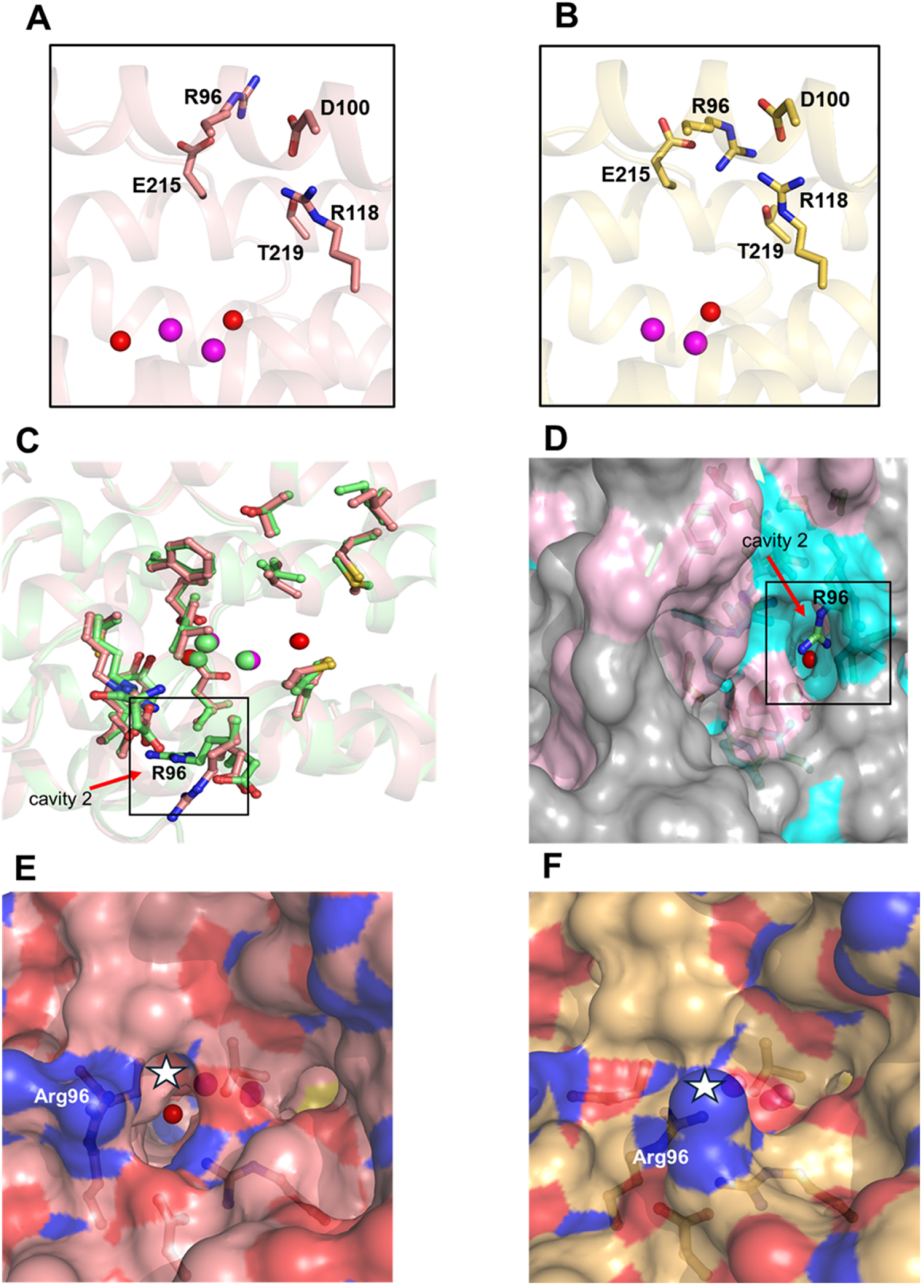
Active site structure of TAO in the absence (A) or presence (B) of compound **1**. (A) The active site in the absence of **1** (PDB ID: 9KUN). The key amino acids are shown as sticks and labeled. Fe atoms and OH^–^ ions are shown as purple and red spheres, respectively. (B) The active site structure in the presence of **1** (PDB ID: 9M2A). Note an ∼70° inward conformational change of R96. (C) Superposed structures of the cartoon models for the ligand-free TAO and TAO–**1** complex showing the active site amino acids (PDB ID: 9KUN and 9M2A). Amino acid residues in the catalytic site are shown in the ball and stick model; those for the ligand-free form are colored pink and the TAO–**1** is colored green. (D) Structures of the ligand-free form in the surface model superposed with the cartoon model of the TAO–**1** structure, with the amino acids shown as sticks (PDB ID: 9KUN and 9M2A). (E) Surface model of the structure of ligand-free TAO (PDB ID: 9KUN). The active site cavity is marked with a black-contoured white star ☆. (F) Surface models of structures of TAO–**1** (PDB ID: 9M2A). The active site cavity, marked with a black-contoured white star ☆, was blocked.

#### Small Structural Changes in the TAO–**1** Complex vs the Ligand-Free TAO Enzyme are Responsible for the Closure of the Active Site Cavity.

Comparing the structure of the TAO–**1** complex (PDB ID: 9M2A) with that of the ligand-free TAO (PDB ID: 9KUN) revealed structural changes that give insight into the inhibitory mechanism of the inhibitor against TAO, which corroborate the inhibition kinetics data. We compared the structure of both forms of the enzyme and found the structural conformations to be largely the same all over the molecule, except at the active site. Figure 6A shows the active site in the ligand-free form to be in an open conformation, while upon binding of compound **1**, the active site of TAO assumes a closed conformation (Figure 6B). Superposing the two substructures (Figure 6C) revealed that the conformational change at the active site was due to the inward flipping of Arg96 side chain into active site cavity 2 (Figure 6D). The cavity was open when **1** was not bound (Figure 6E). Upon binding the inhibitor, the conformational change results in the closure of the active site cavity (Figure 6F). The cavity has been described to be a ubiquinol substrate binding site and the oxygen trough.^60^

### Multiple Sequence Alignment of *T. congolense* AOX and *T. b. brucei* AOX Reveals Similar Secondary Structure Signatures with Over 80 Percent Homology

In an attempt to interrogate the slight variation in the *in vitro* activity of compound **1** against *T. congolense* relative to *T. brucei* and related species, we carried out multiple sequence alignment, which revealed that *T. congolense* AOX has similar secondary structure signatures (α-helices and turns) as reported for *T. b. brucei* AOX.^60,62^ The identity between *T. congolense* AOX and that of *T. b. brucei* is over 80% and has no insertions, but does feature a deletion of the C-terminal hexapeptide NVNKHV, which is not involved in the functionality of the enzyme (Figure 7). In addition, all compound **1**-binding amino acid residues are conserved except for the changes of S140 and V199 in *T. b. brucei*, mostly to K140 and I199 in the other species (Figure 7).

**Figure 7:**
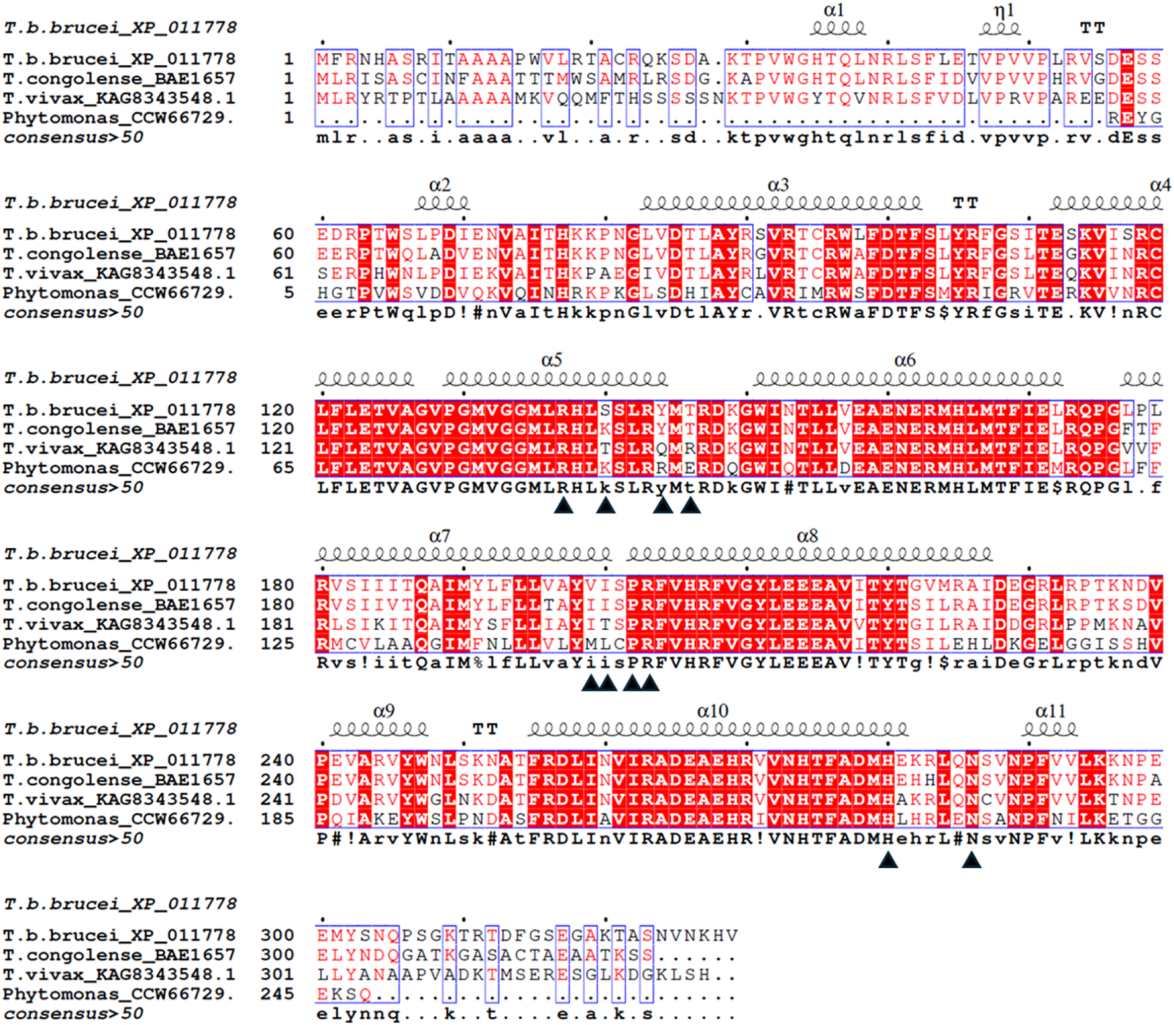
Protein sequence alignment of AOXs. The alignment was created with ESPript 3.0 alignment editor.^63^ The protein sources and their NCBI accession numbers are indicated. The predicted secondary structure elements identified in *T. b. brucei* are shown above the alignment: arrows (β-strands), TT (β-turns), coils (α-helices), and η (3_10_-helix). Amino acid residues shown in white with a red background shade are the residues conserved among AOXs. Amino acid residues implicated for binding compound **1** in *T. b. brucei* AOX are indicated by black triangles. All amino acids binding compound **1** are conserved in *T. congolense* except for the change of S140 and V199 in *T. b. brucei* to K140 and I199 in *T. congolense*.

Importantly, the 10 amino acid residues (R137, S140, Y144, T146, V199, I200, P202, R203, H280, and N286) of *T. b. brucei* AOX that are involved in binding of compound **1** are conserved in *T. congolense* except for S140 and V199 which are changed to K140 and I199, respectively, in *T. congolense* AOX (Figure 7). Structural comparisons of the compound **1** binding sites confirm the structural conservations of V199 and I199 in *T. brucei* AOX and *T. congolense* AOX, respectively. However, the change of S140 to K140 in *T. congolense* AOX resulted in the lysine side chain crashing into the methylene linker position of compound **1** (Figure 8A–C).

**Figure 8:**
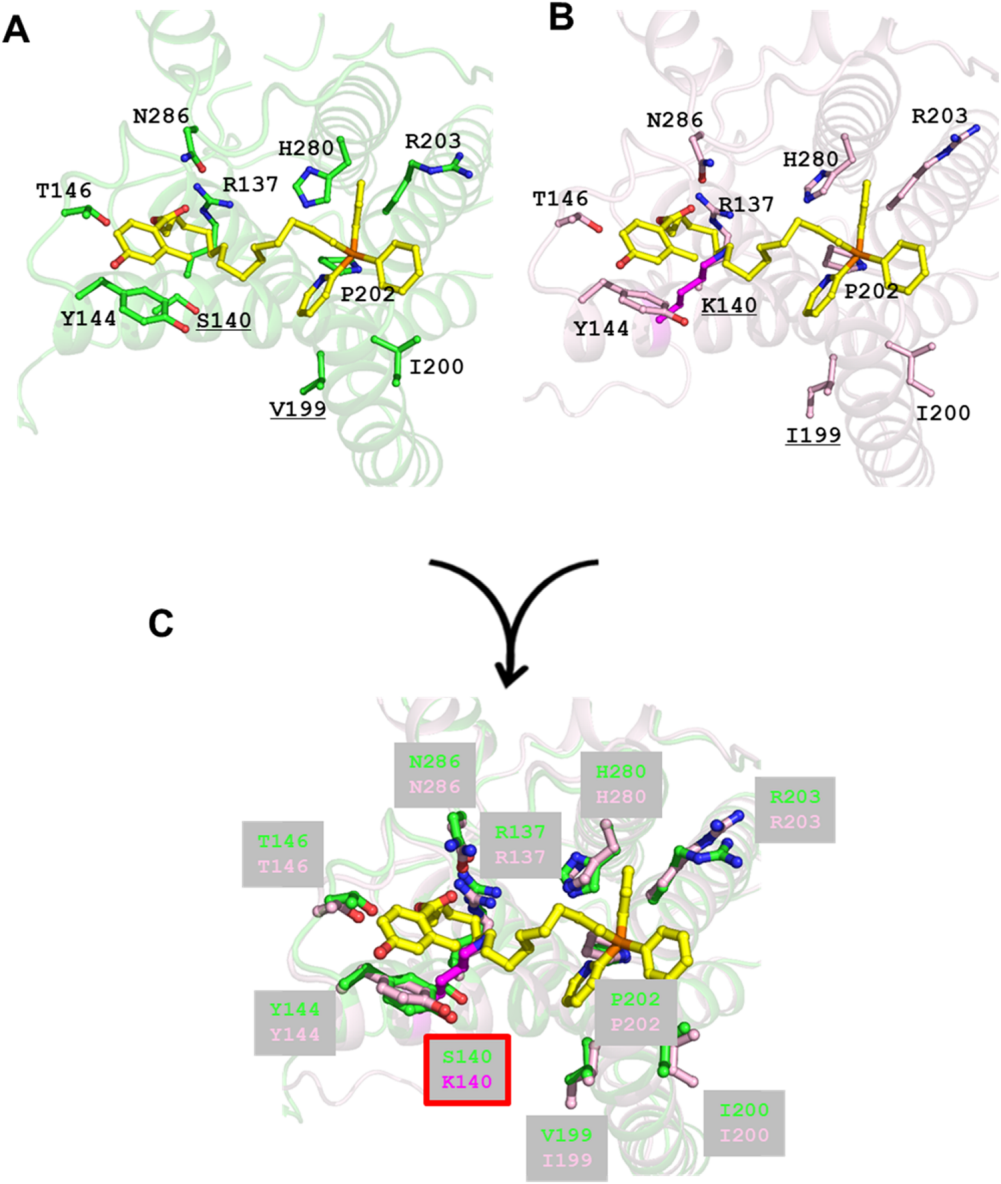
Structures of compound **1** binding sites showing the coordinating residues. (A) Crystal structure of *T. b. brucei* AOX-compound **1** complex (PDB ID: 9M2A). (B) AlphaFold modeled structure of *T. congolense* AOX docked with compound **1**. The structure revealed that the side chain of K140 (underlined) interferes with the binding position of the methylene linker of compound **1**. (C) Superposed structures of *T. b. brucei* (crystal structure, green amino acids) and *T. congolense* (modeled structure, pink amino acids) AOX-compound **1** complexes.

### *In Vivo* Preliminary Study in the STIB795 Mouse Model

To check whether the exceptional nanomolar activities of **1** against both the parasite and the TAO target would translate into in vivo activity, we performed a preliminary study in mice infected with *T. b. brucei* (i.e., STIB795 mouse model). An intraperitoneal (i.p.) dosage of 5 mg/kg/day was chosen based on a pretoxicity study, which showed that **1** was not toxic to uninfected mice at a cumulative dose of 8 mg/kg (i.p.). Considering previous studies with analogs 2-methyl-4-hydroxybenzoate and 2,6-dihydroxy-4-methyl derivatives (**4** and **8**–**9**, respectively),^27^ compound **1** was expected to be stable in serum and toward hepatic metabolism, and the measured stability at 37 °C in PBS buffer was ≥ 1 h. Mice were treated for 4 days with a daily dosage of 5 mg/kg (i.p.). Unfortunately, compound **1** showed no in vivo efficacy at this dose (i.e., did not extend the lifespan of the animals). This was somewhat unexpected because previous nanomolar range benzoate TAO inhibitors **8** and **9** were able to reduce the parasitemia by 95%, 24 h after the last treatment in the STIB900 mouse model (4 × 10 mg/kg ip).^27^ Since the lack of activity could be related to poor pharmacokinetics, we measured the microsomal stability of **1** in mouse (*t*_1/2_ = 4.6 min) and human microsomes (*t*_1/2_ = 6.2 min) showing that this compound was cleared very quickly by hepatic metabolism (CL_int,met_ = 293 ± 19 and 216.1 ± 2.3 μL min^–1^ mg^–1^, respectively). Hence, the difference in in vivo results between **1** and the 2,4-dihydroxy-6-methyl analogs **8** and **9** presumably comes from a different metabolic stability of the latter caused by the presence of an extra OH group in position *ortho* to the benzoate linkage. Although the introduction of an additional OH group can increase the conjugative metabolism, as was shown with analog compound **8** that suffered from phase 2 metabolism in presence of the S9 fraction and cofactor UDPGA (half-live of approximately 2 h), this effect is not totally predictable as shown by the lack of phase 2 metabolism of the quinolin-1-ium analog **9** containing the same 2,4-dihydroxy-6-methyl scaffold.^27^

## Discussion

While sleeping sickness, commonly known as HAT, is a devastating fatal disease in sub-Saharan Africa, there is now a steady decline in its disease burden,^64,65^ although the trend in AAT is not at all favorable, including for the nontsetse-transmitted trypanosomiases.^2^ The idea to tackle trypanosomiasis using the WHO One Health approach is therefore a welcome development.^12,64,66^ This is because by recognizing the intricate interconnection between animal and human health, the One Health approach has the benefit of offering a far-reaching framework for understanding, preventing, and controlling these intertwined HAT and AAT infections, and eventually advancing efforts to eliminate them.

We tested compound **1** on a panel of *in vitro* cultures of trypanosomes causing AAT. Interestingly, the compound was found to be potent against all the causative agents of AAT it was tested against in this work, with similar EC_50_ (i.e., single-digit trypanocidal activities in the low nanomolar range). It is important to note that *T. evansi* and *T. equiperdum* are closely related to *T. b. brucei*. *T. evansi* is derived from *T. b. brucei* via a total loss of maxicircles of mitochondrial DNA in the kinetoplast, which is needed for the parasite to survive as a procyclic form in tsetse flies.^67^ The result is the loss of ability to carry out oxidative phosphorylation,^68^ and the inability to carry out its cycle in tsetse flies, leaving it effectively “trapped” in its long, slender bloodstream form. Though *T. evansi* possesses a minicircle of kinetoplastic DNA, some are akinetoplastic or dyskinetoplastic due to complete loss of the kinetoplastic DNA as observed in some field isolates.^69^ However, some trypanocidal drugs have been reported to induce the dyskinetoplastic forms.^68,70^ *T. equiperdum* is closely related to *T. evansi* and is also reported to be derived from *T. b. brucei* by an alteration of the kinetoplastic DNA, but maxicircles are still present in *T. equiperdum*.^71^ Even though these species of trypanosomes are closely related, the distinction is still clear for the complexity of their minicircle, which is very high in *T. b. brucei* (several hundred classes of minicircle sequences) and very low, if at all present, in *T. evansi* and *T. equiperdum*. This may explain the similarity in activity of the compound on these three *Trypanosoma* species.

As expected from the relative mitochondrial complexity of *T. congolense*, and consistent with our earlier studies,^27^ activity was lower against *T. congolense* with a 13-fold decreased potency relative to *T. b. brucei*. Crucially, even for this species, the in vitro EC_50_ was just 41 ± 7 nM with an SI of 182, delivering a safe and potent compound for animal trypanosomiasis. The variation in the *in vitro* activity of compound **1** against *T. congolense* in comparison with *T. brucei* could be due to the slight difference in the amino acid composition of *T. congolense* TAO around the binding site of compound **1**. Despite of high sequence similarity (>80% identity), *T. congolense* and *T. b. brucei* AOXs differ in the amino acid present at position 140 (serine in *T. brucei* vs lysine in *T. congolense*). The presence of K140 in *T. congolense* AOX results in the lysine side chain crashing into the methylene linker position of compound **1**, possibly resulting in reduced interaction with TAO. However, other species-related differences may also contribute. The reduced sensitivity of *T. congolense* to inhibition by TAO inhibitors in general is probably linked to important differences in the energy metabolism of the bloodstream forms of the two species, which is more complex and much less dependent on glucose in *T. congolense* than in *T. brucei* species^72^ although it still believed to be ultimately dependent on TAO as the sole terminal oxidase.

The results presented in this report indicate that **1** is the most potent (nanomolar) TAO inhibitor among all the phosphonium series we have reported so far.^26–28,43,44^ This has the same range as ascofuranone, an inhibitor of TAO.^36^ Moreover, its allosteric binding position means that, unlike ascofuranone, it does not compete with the natural substrate, ubiquinone, and its activity is independent of substrate concentration.

The effect of the test compound on trypanosome (s427 WT) growth and proliferation was studied after exposure of 24 h. The result of the limited exposure/drug withdrawal experiment showed that the parasites do not recover following drug withdrawal. The drug exposure, even at 2 × EC_50_, had caused irreversible damage at that point and the growth arrest after 4 h and accumulation in G1 phase are consistent with that observation. Moreover, the lipophilic cations are designed (and known) to cross the plasma membrane and mitochondrial membrane and accumulate, ultimately, in the mitochondrion against the concentration gradient. Driven by the differentials in the negatively charged plasma- and mitochondrial transmembrane potentials, lipophilic cations with a dispersed positive charge will rapidly cross these lipid bilayers by an apparently noncarrier-mediated transport mechanism and accumulate significantly and specifically into the mitochondria.^73–76^

The strong accumulation of an active drug linked to a lipophilic cation facilitated by the charged mitochondria enables the inhibition of various essential mitochondrial functions, even with relatively low amounts of extracellular drug concentrations.^29,77,78^ Besides, for the diamidine drugs like pentamidine, diminazene and furamidine, trypanosomes need only to be exposed to the drug for a limited period (about 1–3 h); though these trypanocides acts very slowly, cell death still occurs hours after the cessation of the drug exposure.^79,80^ Like compound **1**, these diamidines are cations and accumulate strongly in the mitochondrion and do not exit the cells upon wash-out.^50,52,81,82^ Indeed, even though pentamidine, a dication, principally enters trypanosomes through an aquaporin,^52^ molecular dynamics modeling has shown that the membrane potential overwhelmingly favors uptake over efflux.^83^ This suggests that even compound **1**’s early effects are effectively irreversible, and trypanosomes are incapable of recovering from an exposure of limited duration. This is important as a short minimum exposure time for a drug reduces the risk of cumulative toxicity associated with keeping the drug at peak plasma concentration levels for longer, as is the case for some of the neutral trypanocides with prolonged administration protocols and serious drug toxicity issues, e.g., nifurtimox, eflornithine, melarsoprol.^84^ Furthermore, for animal trypanosomiasis, a simple administration protocol, preferably a single intramuscular injection, is essential given the paucity of veterinary services in most of the endemic areas and the nomadic lifestyle of many livestock owners.^2,85^

Another major issue associated with existing trypanocides is drug resistance, giving rise to the high priority given to the development of new drugs used for treating trypanosomiasis.^86–88^ Consequently, our test compound was evaluated against a panel of well-characterized multidrug-resistant laboratory strains of *T. b. brucei*, which are known to be resistant to various classes of trypanocides, including the diamidines and melaminophenyl arsenicals. These were used as models to determine whether there is potential for cross-resistance between the test compound and existing classes of trypanocides. This includes a multidrug-resistant strain, B48 that lacks both the *T. brucei* high-affinity pentamidine transporter (HAPT1)/TbAQP2 and the aminopurine transporter TbAT1/P2, which is highly resistant to diamidines and melaminophenyl arsenicals.^53^ The sensitivity of African trypanosomes to two key trypanocides—melarsoprol and pentamidine—is principally mediated by an aquaglyceroporin, TbAQP2, that is associated with water/glycerol transport. TbAQP2 is localized to the flagellar pocket membrane of the parasite,^89^ and it has been shown that chimerization of this channel with aquaglyceroporin 3 (TbAQP3) renders the parasites resistant to both pentamidine and melarsoprol.^52,90,91^ The mechanism of resistance to suramin is not as definitively defined but its mechanism of action appears to involve its uptake through receptor mediated endocytosis^92^ and impact on mitochondrial energy production.^93^

Our findings showed that there were no significant differences in terms of susceptibility between the resistant strains (B48, *aqp1-3 null* and SUR10) and the WT, which implies that cross-resistance with existing first-line AAT and HAT drugs, including diminazene, pentamidine, suramin, melarsoprol, and cymelarsan is unlikely to occur with our test compound. This notwithstanding the fact that the diamidines (pentamidine, diminazene), at least, likewise have mitochondrial targets^81,94^ and suramin changes mitochondrial energy metabolism.^93^ Bearing in mind that resistance to these key trypanocides has been previously associated with the loss of certain cell surface transporters, e.g., the HAPT/TbAQP2 and P2/TbAT1 drug transporters for the diamidines^53,95,96^ and Invariant Surface Glycoprotein (ISG75) for suramin,^92,93^ it is therefore safe to say that the specific surface transporters confirmed to be responsible for drug transport failures in the resistance strains tested in the present research are not associated with the internalization of our test compound. Rather, like the TPP linked compounds, **1** is more likely to diffuse across the trypanosome cell and mitochondria membranes, effectively ruling out any potential cross-resistance issues when the resistance is linked to drug transporters.^97^ Once the test compound accumulates in the mitochondria of trypanosomes it is by design expected to bind to TAO and inhibit its activity. To understand the mechanism of this binding, Lineweaver–Burk plot analysis was carried out on the results of the direct inhibitory studies. The result indicates that **1** binds to TAO in a noncompetitive manner. Neutral benzoate derivatives with large alkyl chains and cationic TAO inhibitors such as **6** (R_1_ = CH_3_, R_2_ = H, LC = quinolin-1-ium) and **8** (R_1_ = CH_3_, R_2_ = OH, LC = TPP) display a competitive inhibition mode.^27^ All these compounds have in common the 4-hydroxybenzoic acid scaffold and the presence of a methyl group in R_1_ (Figure 1). They are expected to bind the alternative oxidase with the ester alkyl chain and the LC group pointing outward from the active site. In contrast, analogs with an unmodified 2,4-DHB scaffold (R_1_ = R_3_ = H, LC = nil) appear to inhibit TAO noncompetitively.^27,31^

In the crystal structure of the rTAO–**1** complex reported here, we show a new noncompetitive mode of binding to an allosteric site of TAO for this class of inhibitors; compound **1** binds on the surface of the protein, far from the active site, and promotes a conformational change (i.e., inward flipping of Arg96 side chain) that blocks the entry of the ubiquinol binding site cavity. Since compound **1** was bound at helices 1 and 4, which form the membrane anchor of TAO, we hypothesize that **1** may further interfere with the membrane binding and functionality of TAO. This noncompetitive mode of inhibition is an important advance, as the inhibitor will not be displaced by a buildup of substrate.

Based on these results, one may wonder if other triarylphosphonium-based TAO inhibitors could have a binding mode to TAO similar to **1**. In the absence of more crystal structures, it is difficult to answer this question. Two other potent noncompetitive benzoate-based TAO inhibitors somewhat related to **1** have been described earlier (IC_50_ = 14 and 6.9 nM, respectively), although no crystal structure is available.^27^ These compounds possess a 2,4-dihydroxybenzoate scaffold, a linker of 16 and 10 methylene units, respectively, but they do not hold a lipophilic TPP or PDPP cationic moiety. Even though these compounds present structural similarities with compound **1**, the lack of crystal structure does not allow to conclude whether they share the same binding mode to TAO. However, the dissimilar in vitro inhibition data of very close structural analogs of **1** (LC = PDPP, R_2_ = H, IC_50_ = 1.3 nM) such as **2** (LC = PDPP, R_2_ = OH, IC_50_ = 1600 nM) and **4** (LC = TPP, R_2_ = H, IC_50_ = 81 nM) points to a very specific interaction of **1** with the allosteric site of TAO, which appears to depend on both the LC structure (“tail group”) and the 4-hydroxybenzoic acid (“head group”) substitution pattern. We observed from the crystal structure that the pyridinyl nitrogen of **1** was involved in multiple hydrogen bond interactions with different residues at the binding site. Therefore, this pyridine N resulted in tighter binding of **1** to TAO, contributing to its higher potency against TAO, and improved killing potential on the trypanosomes. The addition of a 2-OH group in the benzoic acid head of **1** yielded a 1000-fold weaker inhibitor, **2**. In this case, an unfavorable interaction due to OH–OH repulsion with tyrosine Y144 from the allosteric binding site could explain this difference.

These hypotheses should be tested in order to design new potent mitochondrion-targeted inhibitors of TAO. In addition, further SAR studies will be needed to reduce the hepatic metabolism of hit compound **1**. Although nanoformulations^98,99^ have previously been proposed to increase drug efficacy and even bypass resistance mechanisms in *T. brucei* (*e.g.*, packaging of pentamidine in chitosan-coated nanoparticles),^100^ a chemical modification of the benzoate group to reduce ester hydrolysis kinetics might be a more convenient (and cheaper) strategy to increase its effectivity against African trypanosomiasis.

## Conclusions

In this work, we report the discovery of an extremely potent 2-pyridinyldiphenylphosphonium-derived TAO inhibitor (**1**) with low nanomolar activity against wild-type and drug-resistant *T. brucei* strains. Hit compound **1** displayed broad activity against important African trypanosome pathogens (*T. evansi*, *T. equiperdum*, *T. congolense*). We showed by direct inhibition, kinetic and crystallographic studies of a **1**-TAO complex, a novel mode of inhibition of TAO that could be exploited to design new drugs against AAT and HAT. Apart from allosterically closing the access to the ubiquinol binding site, the binding of **1** may interfere with the functionally essential TAO attachment to the inner mitochondrial membrane, as the allosteric site is formed by helices 1 and 4, which form the membrane anchor of TAO. Our findings suggest that further SAR or nanoformulation to reduce the hepatic metabolism should lead to the development of a new class of broad-spectrum trypanocidal compounds against African trypanosomiasis.

## Experimental Part

### Chemistry

Anhydrous acetonitrile was purchased to Aldrich/Fluka in SureSeal bottles and used as received. Thin Layer chromatography (TLC) was performed on silica gel 60 F254 aluminum TLC plates (MERCK). Silica chromatography was performed on a FlashMaster Personal system using FlashPack SI prepacked columns. LC-MS spectra were recorded on a WATERS apparatus integrated with an HPLC separation module (2695), PDA detector (2996), and Micromass ZQ spectrometer using electrospray ionization in positive mode (ESI^+^). Analytical HPLC was performed with a SunFire C18–3.5 μm column (4.6 mm × 50 mm). Mobile phase A: CH_3_CN + 0.08% formic acid and B: H_2_O + 0.05% formic acid. UV detection was carried over 190 to 440 nm. Accurate mass was measured with an Agilent Technologies Q-TOF 6520 spectrometer using electrospray ionization. ^1^H NMR and ^13^C NMR spectra were registered on a Bruker Avance-300 and Bruker-400 spectrometers. Chemical shifts of the ^1^H NMR spectra were referenced to the residual proton resonance of CDCl_3_ (δ 7.26 ppm) or CD_3_OD (δ 3.31 ppm). Chemical shifts of the ^13^C NMR spectra were referenced to CDCl_3_ (δ 77.16 ppm) or CD_3_OD (δ 49.0 ppm). Coupling constants (*J*) are expressed in hertz (Hz). All the biologically tested compounds were ≥95% pure by HPLC. The synthesis and rTAO inhibition of **3**,^46^ **4**,^27^ **5**,^46^ **6**,^27^ **8**,^27^ **9**,^27^ **10**,^26^ **11**,^46^ and **12** ^28^ has been reported before.

#### {14-[(4-Hydroxy-2-methylbenzoyl)­oxy]­tetradecyl}­diphenyl­(pyridin-2-yl)­Phosphonium Bromide (**1**)

A Kimax tube was charged with **13** (35.1 mg, 0.082 mmol) and diphenyl­(2-pyridinyl)­phosphine (22.7 mg, 0.086 mmol) in anhydrous acetonitrile (1 mL). The solution was stirred 6 days at 80 °C under argon atmosphere. The solvent was removed under vacuum and the crude residue was purified by flash chromatography (2 g SI cartridge) with CH_2_Cl_2_/MeOH (100:0 → 95/5) to yield **1** as brownish foam (22 mg, 39%). HPLC (UV) > 95%. LRMS (ESI^+^) *m*/*z* = 610 (M + H)^+^. ^1^H NMR (300 MHz, CDCl_3_) δ 9.00 (s, 1H), 8.86 (d, *J* = 4.8 Hz, 1H), 8.33 (brt, *J* = 7.1 Hz, 1H), 8.18–8.05 (m, 1H), 7.91–7.73 (m, 6H), 7.73–7.59 (m, 6H), 6.92 (s, 1H), 4.26 (t, *J* = 5.6 Hz, 2H), 3.69–3.54 (m, 2H), 2.52 (s, 3H), 1.78–1.67 (m, 2H), 1.65–1.01 (m, 22H). ^13^C NMR (75 MHz, CDCl_3_) δ 167.7, 161.1, 152.1 (d, *J* = 19.0 Hz), 145.3, 143.8, 142.8, 138.7 (d, *J* = 10.2 Hz), 135.3 (d, *J* = 3.6 Hz), 133.9 (d, *J* = 9.8 Hz), 132.9, 131.7 (d, *J* = 24.1 Hz), 130.6 (d, *J* = 12.4 Hz), 128.3 (d, *J* = 3.8 Hz), 120.3, 118.9, 117.5 (d, *J* = 85.3 Hz), 113.4, 64.3, 30.6 (d, *J* = 15.7 Hz), 29.7, 29.5, 29.4, 29.3, 29.2, 29.1, 28.8, 28.7, 26.1, 22.4, 22.2 (d, *J* = 49.3 Hz). ^31^P NMR (162 MHz, CDCl_3_) δ +20.56. HRMS (ESI^+^) *m*/*z* 610.3463 (C_39_H_49_NO_3_P requires 610.3450).

#### {14-[(2,4-Dihydroxy-6-methylbenzoyl)­oxy]­tetradecyl}­diphenyl­(pyridin-2-yl)­Phosphonium Bromide (**2**)

A Kimax tube was charged with **14** (17.2 mg, 0.039 mmol) and diphenyl­(2-pyridinyl)­phosphine (10.2 mg, 0.039 mmol) in anhydrous acetonitrile (0.7 mL). The solution was stirred for 5 days at 80 °C under an argon atmosphere. The solvent was removed under vacuum to give a crude brown oil, which was dissolved in CH_2_Cl_2_ and treated with hexane until a precipitate appeared. The flask was stored in the fridge overnight. The supernatant was discarded, and the brown gummy precipitate was dissolved in MeOH (0.5 mL) and treated with Et_2_O until precipitation started. The flask was allowed to stand in the fridge for 2 weeks, whereupon a beige solid settled at the bottom of the flask. The supernatant was discarded and the solid was rinsed with Et_2_O and dried under vacuum. Beige solid (11.2 mg, 41%). HPLC (UV) > 95%. LRMS (ESI^+^) *m*/*z* = 626 (M + H)^+^. ^1^H NMR (400 MHz, CDCl_3_) δ 11.75 (s, 1H), 8.87 (d, *J* = 4.8 Hz, 1H), 8.28 (t, *J* = 6.8 Hz, 1H), 8.11 (tdd, *J* = 7.7, 5.1, 1.8 Hz, 1H), 7.90–7.75 (m, 6H), 7.74–7.59 (m, 5H), 6.66 (d, *J* = 2.5 Hz, 1H), 6.34 (d, *J* = 2.5 Hz, 1H), 4.32 (t, *J* = 5.8 Hz, 2H), 3.66–3.54 (m, 2H), 2.46 (s, 3H), 1.74 (p, *J* = 6.5 Hz, 2H), 1.67–1.39 (m, 6H), 1.37–1.06 (m, 16H). ^13^C NMR (126 MHz, CDCl_3_) δ 172.3, 165.5, 162.9, 152.0 (d, *J* = 19.1 Hz), 144.7 (d, *J* = 116 Hz), 143.2, 138.7 (d, *J* = 10.1 Hz), 135.2 (d, *J* = 3.2 Hz), 134.0 (d, *J* = 9.5 Hz), 131.9 (d, *J* = 24.1 Hz), 130.5 (d, *J* = 12.8 Hz), 128.2 (d, *J* = 3.7 Hz), 117.7 (d, *J* = 85.3 Hz), 112.5, 104.3, 101.6, 65.1, 30.6 (d, *J* = 15.7 Hz), 29.5, 29.4, 29.2, 29.1, 28.8, 28.7, 28.5, 28.3, 25.9, 24.6, 22.6 (d, *J* = 4.7 Hz), 22.2 (d, *J* = 49.1 Hz). ^31^P NMR (162 MHz, CDCl_3_) δ +20.53. HRMS (ESI^+^) *m*/*z* 626.3407 (C_39_H_49_NO_4_P requires 626.3399).

#### {14-[(3,5-Dichloro-2,4-dihydroxy-6-methylbenzoyl)­oxy]­tetradecyl}­Triphenylphosphonium Bromide (**7**)

Under an inert atmosphere, **16** (9.2 mg, 18 μmol) and PPh_3_ (4.8 mg, 18.3 μmol) were suspended in anhydrous CH_3_CN (1.5 mL). Thereafter, the mixture was heated up at 80 °C and kept under stirring for 48 h. After that, the solution was cooled down at room temperature and the solvent evaporated under high vacuum. The remaining oily solid was washed with hot Et_2_O and the organic solvent was discarded, yielding **7** as a brownish gummy solid (8.7 mg, 62%). HPLC (UV) > 95%. ^1^H NMR (300 MHz, CD_3_OD) δ 1.41–1.81 (m, 24H), 2.53 (s, 3H), 3.35–3.38 (m, 2H), 4.38 (t, *J* = 6.5 Hz, 2H), 7.73–7.89 (m, 15H). ^13^C NMR (125.75 MHz, CD_3_OD) δ 171.7, 157.2, 155.1, 137.0, 136.3 (d, *J* = 3.0 Hz), 134.8 (d, *J* = 9.9 Hz), 131.51 (d, *J* = 12.6 Hz), 120.0 (d, *J* = 86.3 Hz), 115.9, 108.6, (C_5_ not seen), 67.2, 31.7, 31.5, 30.6, 30.5, 30.5, 30.4, 30.4, 30.3, 30.1, 29.9, 29.5, 27.1, 23.5 (d, *J* = 4.3 Hz), 22.7 (d, *J* = 51.1 Hz), 19.5. HRMS (ESI^+^) *m*/*z* 693.2667 (C_39_H_49_NO_4_P requires 693.2662).

#### 14-Bromotetradecyl 4-Hydroxy-2-Methylbenzoate (**13**)

The reaction was performed in a Kimax tube with a screw cap. A mixture of 4-hydroxy-2-methyl benzoic acid (67 mg, 0.44 mmol), 1,14-dibromotetradecane^45^ (157.6 mg, 0.44 mmol) and sodium bicarbonate (37 mg, 0.44 mmol) in anhydrous acetonitrile (4.5 mL) was stirred 9 days at 65 °C. The solvent was removed under vacuum and the crude residue was dissolved in dichloromethane. The nonsoluble inorganic salts were filtered off and the filtrate was evaporated under vacuum. Flash chromatography (5 g silica cartridge, dry load) with hexanes/EtOAc (100:0 → 75:25) yielded **13** as a colorless solid (73 mg, 39%). HPLC (UV) = 94%. LRMS (ESI^+^) *m*/*z* = 427 (M + H)^+^. The spectroscopic data were congruent with the reported ones.^27^

#### 14-Bromotetradecyl 2,4-Dihydroxy-6-Methylbenzoate (**14**)

This compound was synthesized following the same protocol starting from 2,4-dihydroxy-2-methyl benzoic acid.^27^

## Biology

### Parasites and Cultures

Several different strains of *T. b. brucei* (BSF) were used in this study: (1) Wild type *T. b. brucei*, strain Lister 427 (s427; MiTat 1.2/BS221); (2) A multidrug-resistant strain, B48 which was created from s427-WT after deletion of both the TbAT1/P2 drug transporter and in vitro adaptation to pentamidine, leading to the functional loss of the high affinity pentamidine transporter (HAPT1);^53^ (3) the pentamidine-resistant Aquaporin 1–3 triple null (*aqp1-3* null strains), which deleted all aquaporin genes from *T. b. brucei* ^56^ including TbAQP2, which was specifically implicated in pentamidine–melarsoprol cross-resistance in field isolates.^90^ Other *Trypanosoma* species used in this study include *T. evansi* AntTat 3/3 (Wild-type);^100^ and *T. equiperdum* BoTat 1 (Bordeaux trypanozoon antigenic type 1) P (wild-type).^101^ All the trypanosomes were used only as bloodstream trypomastigotes and cultured in the standard Hirumi’s Modified Iscove’s medium #9 (HMI-9), supplemented with 10% heat-inactivated Fetal Bovine Serum (FBS), 14 μL/L β-mercaptoethanol, and 3.0 g sodium hydrogen carbonate per liter of medium (pH 7.4). Trypanosomes were cultured in vented flasks at 37 °C in a 5% CO_2_ atmosphere.^102^

### Resazurin-Based Drug Sensitivity Assay

The susceptibilities of trypanosomes to compound **1** were determined as previously described using the resazurin (Alamar Blue) assay.^25^ Briefly, this protocol involves preparing a 96-well cell culture plate with a 100 μL serially diluted test compound (200 μM top concentration) in HMI-9 + 10% FBS (24 wells per control drug or test compound, with the 24th well serving as drug-free). This is followed by adjusting the cell density of mid-log-phase trypanosome cultures to the required concentration of 2 × 10^5^ cells/mL (2 × 10^4^ cells/well). Then, 100 μL of the adjusted cell density/culture was added to all 24 wells in the plate, then incubating trypanosomes and test compound for 48 h followed by the addition of 20 μL filter-sterilized resazurin solution prepared by adding 25 mg resazurin sodium salt to 200 mL filter-sterilized PBS. This was followed by a further 24 h of incubation. All incubations were carried out at 37 °C with 5% CO_2._ Standard drugs were used as positive control, where appropriate, including pentamidine isothionate, diminazene aceturate, or SHAM. Fluorescence was measured in the 96-well plates with a FLUOstar Optima (BMG Labtech, Durham, NC, USA) at wavelengths of 544 nm for excitation, 590 for emission, and a gain of 1250. EC_50_ values were calculated using GraphPad Prism 9.0 software via nonlinear regression with an equation for a sigmoidal dose–response curve with variable slope (GraphPad Software Inc., San Diego, CA, USA).

### Cytotoxicity Assay Using Human Embryonic Kidney (HEK) 293 Cells

The toxicity of **1** was evaluated in mammalian cells according to a method previously described.^102^ In brief, HEK cells were grown in a culture made of 500 mL Dulbecco’s Modified Eagle’s Medium (DMEM) (Sigma), 50 mL New-born Calf Serum (NBCS) (Gibco), 5 mL l-Glutamax (200 mM, Gibco), and 5 mL Penicillin/Streptomycin (Gibco). HEK cells were incubated in vented flasks at 37 °C/5% CO_2_ and were passaged upon reaching 80–85% confluence. For the assay, cells were suspended at a density of 3 × 10^5^ cells/mL, and then 100 mL of it was added to each well of a 96-well plate (24 wells per control drug or test compound). The plate was incubated at 37 °C/5% CO_2_ for 24 h to allow cytoadhesion to the bottom of the plate. Following this, serial drug dilutions (400 μM top concentration; 150 μL/well) were prepared in a separate sterile plate, with the 24th well serving as drug-free. Then, 100 mL of these drug dilutions were transferred to the corresponding plate wells containing the HEK cells. PAO was used as the positive control. The plate was then incubated at 37 °C/5% CO_2_ for a further 30 h, followed by adding 10 mL of filtered-sterile resazurin solution (125 mg/L in PBS). This was followed by a final incubation for 24 h at 37 °C/5% CO_2_. The plate was then read in a FLUOstar OPTIMA fluorimeter set at a wavelength of 530 nm for excitation and 590 nm for emission. The data obtained were analyzed using GraphPad Prism 9.0 (GraphPad Software Inc., San Diego, CA, USA) to determine the CC_50_ values. The selectivity index was calculated as CC_50_ (HEK)/EC_50_ (*Trypanosoma*).

### Assessment of Cell Cycle Progression in *T. brucei* Using Flow Cytometry

DNA content in *T. b. brucei* bloodstream forms was performed essentially as described.^77^ Briefly, 1 mL of compound **1**-treated (2 × EC_50_) or nontreated (control) culture, containing approximately 1–2 × 10^6^ cells, was centrifuged at 1620*g* for 10 min at 4 °C, washed once in PBS containing 5 mM of EDTA and resuspended and fixed in 1 mL of 70% methanol and 30% PBS/EDTA. The tube with the cells was left at 4 °C overnight in the dark, and the samples were subsequently washed once with 1 mL PBS/EDTA, resuspended in 1 mL PBS/EDTA containing 10 μg/mL propidium iodide and incubated at 37 °C for 45 min. RNase A (10 μg/mL) was added before the samples were analyzed on a Becton Dickinson FACS Calibur using the FL2-Area detector and CellQuest software. The data obtained were analyzed using FlowJo software (FlowJo LLC, Ashland, OR, USA).

### Assessment of DNA Configuration Using Fluorescence Microscopy

The configuration of nuclei and kinetoplast DNA of treated and untreated *T. brucei* was visualized using a Vectashield mounting medium with the dye 4,6-diamidino-2-phenylindole (DAPI) (Vector Lab. CA, USA), as described.^103^ Briefly, 1 × 10^7^ cells/mL were treated with 2 × EC_50_ of compound **1** and the untreated culture served as control. The 1 mL of the cultures were collected after the specified incubation period, fixed in 4% paraformaldehyde in PBS for 10 min at room temperature, DAPI treated, and the slides were then viewed under UV light on a Zeiss Axioskop 2 fluorescent microscope (Carl Zeiss Microscopy, USA) using Hamamatso digital camera and Openlab software. At least 500 cells were scored per slide for the number of kinetoplasts (K) and nuclei (N) as 1N1K, 1N2K, 2N2K, or other.

### TAO Inhibition and Inhibitory Kinetics Assay

As previously described, the Ubiquinol oxidase activity measurement was carried out using recombinant TAO lacking the mitochondrial targeting signal (ΔMTS).^27^ The assay involves recording the change in absorbance of the substrate, ubiquinol-1 (ε_278_ = 15,000M^−1^ cm^−1^), at 278 nm in the presence or absence of the test/control compound. The reaction was carried out in a 1 cm cuvette for 2 min using a V-630 Jasco UV–vis spectrophotometer (Jasco Corporation, Tokyo, Japan). Briefly, the procedure involves preincubating 250 ng of the enzyme for 2 min in a 50 mM Tris-HCl (pH 7.3) buffer containing the detergent octaethylene glycol monododecylether (0.05% (w/v)) in a total reaction volume of 1 mL at 25 °C. The reaction was initiated by the addition of 150 μM final concentration of ubiquinol-1 to the cuvette. For the inhibition reaction, 250 ng of the enzyme was preincubated with varying inhibitor concentrations for 2 min using the same buffer before the substrate was added. Ascofuranone and SHAM were used as positive controls, while an equal volume of DMSO was used as a negative control; the DMSO used did not affect the activity of TAO. Also, background activity measurements were conducted throughout the experiment to confirm that there was no auto-oxidation of ubiquinol-1 in the medium. This was achieved using the same protocol but without TAO. IC_50_ of **1** was determined at a fixed amount of the TAO and varying concentrations of **1**. The residual activities were plotted against the corresponding inhibitor concentration in a GraphPad Prism-9 software to obtain the IC_50_ value. The TAO kinetic determination was carried out by performing TAO assay in the presence of varying concentrations (0, 5, 10, and 15 nM) of **1** and with varying substrate (Ubiquinol; Q_1_H_2_) concentrations (0–600 μM).

#### Microsomal Stability Assays

Pooled mixed gender human liver microsomes (HLMs) and Pooled CD1 mouse liver microsomes (female) were purchased from Tebubio. The NADPH regenerating system was purchased from Promega. All other reagents and solvents were of special or analytical grade and were commercially available. The assays were performed according to a reported protocol^104^ with small changes. Briefly, the test compound **1** (C = 1 μM) was preincubated with the NADPH regenerating system in a thermoblock with constant agitation (750 rpm) for 5 min at 37 °C in 0.1 M phosphate buffer, pH 7.4. The reactions were initiated by adding liver microsomes at a final concentration of 0.5 mg/mL in a total volume of 250 μL. After 0, 10, 15, 30, and 60 min incubations at 37 °C with constant agitation (750 rpm), the reactions were stopped by adding 250 μL of cold (−20 °C) acetonitrile. The samples were sonicated for 5 min and then centrifuged 5 min at 10,000*g*, 4 °C. The supernatants were analyzed with LC/MS for the amount of parent compound remaining, and the CL_int_ and *t*_1/2_ were determined.

#### *In Vivo* STIB795 Mouse Model

The efficacy assay was performed as reported^105^ with eight NMRI mice infected with bloodstream forms of *T. b. brucei* STIB795/luc strain expressing the red-shifted luciferase gene.^106^ Briefly, 3 days postinfection, compound **1** (5 mg/kg) was administered intraperitoneally as a 10% DMSO solution in water for 4 days (qd). The treated group (five mice) and the untreated control group (3 mice) were monitored for blood parasitemia over 14 days. At this time, all mice were found positive for parasites. *In vivo* efficacy studies in mice were conducted at the Swiss Tropical and Public Health Institute (Basel) (License number 2813), according to the rules and regulations for the protection of animal rights (“Tierschutzverordnung”) of the Swiss “Bundesamt für Veterinärwesen”. They were approved by the veterinary office of Canton Basel-Stadt, Switzerland.

### Preparation of TAO–**1** Complex Crystal

The alternative oxidase from *T. brucei* was crystallized according to the method described previously^60^ using 28–34% (w/v) PEG 400, 100 mM imidazole buffer pH 7.4, and 500 mM potassium formate as the reservoir solution. The crystals of the TAO-inhibitor complex were prepared by soaking TAO crystals in the cryo-protectant solution [50% (w/v) PEG 400, 500 mM potassium formate and 100 mM imidazole buffer pH 7.4] supplemented with compound **1** (1 mM) for 90 min at 20 °C. The crystals were mounted in a nylon loop and flash-frozen in a stream of gaseous nitrogen at 100 K.

### Data Collection and Structural Refinement

The diffraction data of the TAO–**1** complex crystal was collected at 3.01 Å resolution at 100 K at KEK-PF BL17A (Tsukuba, Japan). The data set was processed and scaled with HKL2000.^107^ The crystal belongs to the orthorhombic space group C2 with unit cell parameters: *a* = 151.4, *b* = 221.7, *c* = 62.9 Å, and β = 114°. In the crystal structure, there were four monomers in the asymmetric unit. The initial model of TAO–**1** complex was determined by molecular replacement (MR) using the CCB-TAO complex without the inhibitor [PDB code: 3W54;^60^] as a search model. The program, Phaser^108^ in CCP4i was used for MR. Manual rebuilding and crystallographic refinement of all structures were performed using the COOT^109^ and REFMAC5.^110^ The structure was refined by amplitude-based twin-refinement in REFMAC5 ^110^ to final *R*_work_/*R*_free_ values of 0.195/0.254. On average, about 30 residues of N- and C-termini of TAO were missing as a result of flexibility. The inhibitor (**1**) is located far away from the active center, and one molecule is bound in the asymmetric unit (four subunits). Data collection and structural refinement statistics are summarized in Supporting Information (Table S1). Figures showing protein structures were prepared with the graphics program PyMOL (http://www.pymol.org/).

The atomic coordinates and structure factors have been deposited in the Protein Data Bank, www.pdb.org, with PDB codes 9M2A (TAO–**1** complex) and 9KUN (unbound ligand-free TAO), respectively.

### Multiple Sequence Alignment of Kinetoplastid AOXs and AI-Based Structural Modeling of *T. congolense* AOX

Amino acid sequences of AOXs from *T. b. brucei*, *T. congolense*, *T. vivax*, and *Phytomonas*, with accession codes XP_011778038.1, BAE16577.1, KAG8343548.1, and XP_009313914.1, respectively, were obtained from the NCBI database (https://www.ncbi.nlm.nih.gov/protein). The sequences were compared by multiple sequence alignment generated using the CLUSTAL W program,^111^ and sequence alignment was generated using ESPript 3.0.^63^ The predicted secondary structure elements and compound **1** inhibitor binding amino acids were determined and annotated. For structural modeling, amino acid sequence of the *T. congolence* AOX was subjected to the deep learning algorithm AlphaFold2.^112^ The modeled 3-D structure was visualized and processed with Pymol program.^113^

## Supplementary Material





## Notes

^1^H NMR spectra and HPLC-MS traces of compounds **1, 2**, and **7**; PDB ID of new crystal (X-ray) structures: TAO-**1** complex (9M2A) and ligand-free TAO (9KUN). Authors will release the atomic coordinates upon article publication (PDF)
Molecular Formula Strings and biological data of compounds **1**–**12** (CSV)

## Abbreviations Used

AAT: Animal African Trypanosomiasis
BSF: bloodstream form
2,4-DHB: 2,4-dihydroxybenzoate
FBS: fetal bovine serum
HAT: human African trypanosomiasis
LC: lipophilic cation
PDPP: 2-pyridinyldiphenylphosphonium
RF: resistance factors
rTAO: recombinant TAO enzyme
SHAM: 2,4-salicylhydroxamate
TAO: trypanosome alternative oxidase
TPP: triphenylphosphonium
WHO: World Health Organization
