# Supplementary material for: Uncovering the Unusual Inhibition Mechanism of a Trypanosome Alternative Oxidase Inhibitor Displaying Broad-Spectrum Activity against African Animal Trypanosomes
Source: J Med Chem. 2025 Jun 4;68(16):17155–74. doi: 10.1021/acs.jmedchem.5c00631 (PMC12406258; doi:10.1021/acs.jmedchem.5c00631)

Supporting Information

Uncovering the unusual inhibition mechanism of a trypanosome alternative oxidase inhibitor displaying broad-spectrum activity against African Animal Trypanosomes

Godwin U. Ebiloma[1,2], Emmanuel O. Balogun[3,4,5,] Natsumi Arai[2], Momoka Otani[2], Cecilia Baldassarri[6], Amani Alhejely[7], Eduardo Cueto-Díaz[8], Harry P. De Koning[9], Christophe Dardonville[8*], Tomoo Shiba[2*]

[1]School of Science, Engineering & Environment, University of Salford, Manchester M5 4NT, United Kingdom.

[2]Graduate School of Science and Technology, Department of Applied Biology, Kyoto Institute of Technology, Kyoto 606-8585, Japan.

[3]Department of Biochemistry, Ahmadu Bello University, Zaria 2222, Nigeria

[4]Department of Biomedical Chemistry, Graduate School of Medicine, The University of Tokyo, Tokyo 113-0033, Japan

[5]Center for Discovery and Innovation in Parasitic Diseases, Skaggs School of Pharmacy and Pharmaceutical Sciences, University of California San Diego, 9500 Gilman Drive, La Jolla, CA 92093, USA.

[6]Medicinal Chemistry Unit, School of Pharmacy, Chemistry Interdisciplinary Project (ChIP), University of Camerino, Via Madonna delle Carceri, 62032 Camerino, Italy.

[7]Biology Department, Darb University College, Jazan University, Jazan 82817-2820, Saudi Arabia.

[8]Instituto de Química Médica, IQM–CSIC, Juan de la Cierva 3, E–28006 Madrid, Spain.

[9]School of Infection and Immunity, College of Medical, Veterinary and Life Sciences, University of Glasgow, Glasgow G43 2DX, United Kingdom.

*Corresponding authors: Christophe Dardonville (dardonville@iqm.csic.es)

Tomoo Shiba (tshiba@kit.ac.jp)

Contents: Page

1) Table S1: data collection and processing for TAO–**1** complex (9KBV) and ligand-free TAO (9KUN). S2

2) NMR spectra and HPLC-MS traces of compounds **1**, **2**, and **7**. S3

Table S1. Data collection and processing. X-ray diffraction data were collected for crystals obtained in the presence (TAO–**1** complex) and absence (ligand-free TAO) of compound **1**.

	TAO–**1** complex	TAO (Ligand-free open)
Diffraction source	KEK BL-17A	SPring-8 BL44XU
Wavelength (Å)	0.98000	0.90000
Temperature (K)	100	100
Space group	*C*2	*C*2
a, b, c (Å)	151.4, 221.7, 62.9	149.4, 223.0, 62.9
α, β, γ (°)	90, 114.5, 90	90, 115.3, 90
Resolution range (Å)	50-3.01 (3.05-3.01)	50-2.70 (2.75-2.70)
Total No. of reflections	96,885	142,739
No. of unique reflections	35,302	48,896
Completeness (%)	92.0 (80.9)	98.2 (96.8)
Multiplicity	2.8 (2.4)	2.8 (2.6)
$\langle I/\sigma(I) \rangle$	6.3 (1.1)	8.0 (1.3)
$CC_{1/2}$ (in high resolution bin)	0.785	0.777
R_{merge}	0.071 (0.673)	0.066 (0.772)
Refinement		
Resolution (Å)	20-3.01	20-2.70
used reflections	28,991	41,562
R_{work} / R_{free}	0.195 / 0.254	0.207 / 0.258
RMSD		
Bond length (Å)	0.010	0.013
Bond angle (°)	1.619	1.767
PDB code	9KBV	9KUN

Compound **1**

^{1}H NMR ($CDCl_3$, 300 MHz)

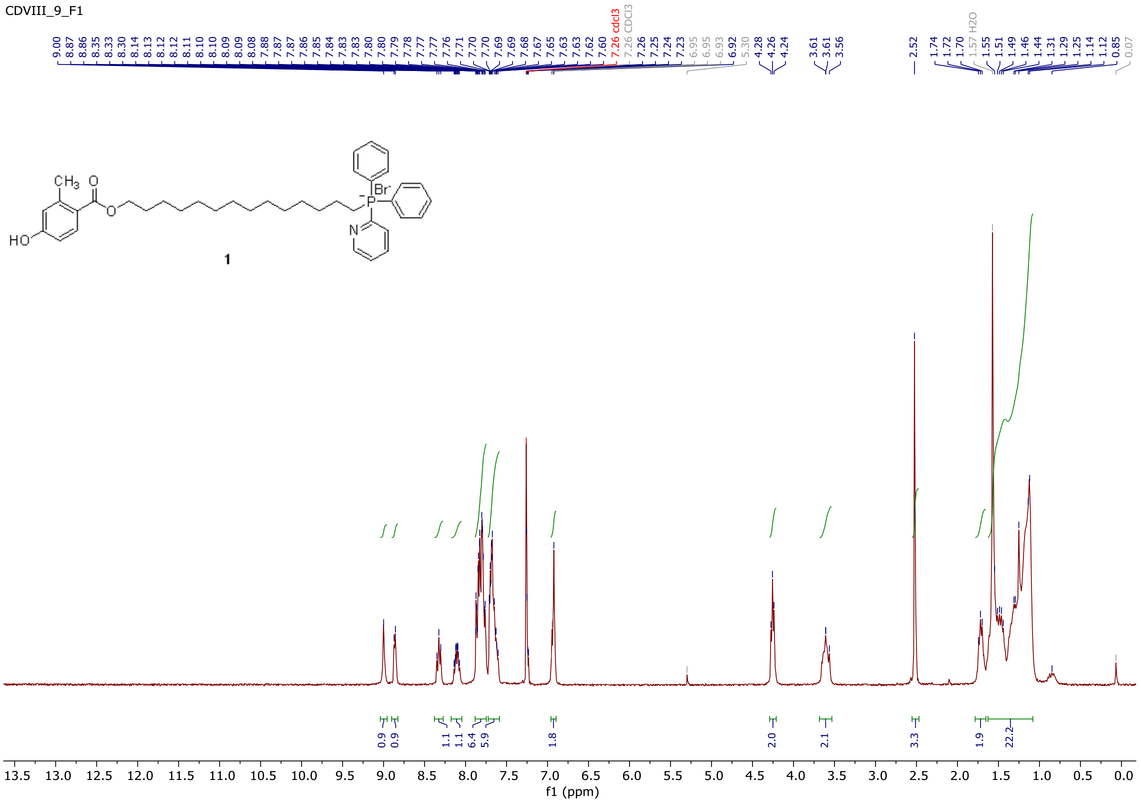

^{13}C NMR ($CDCl_3$, 75 MHz)

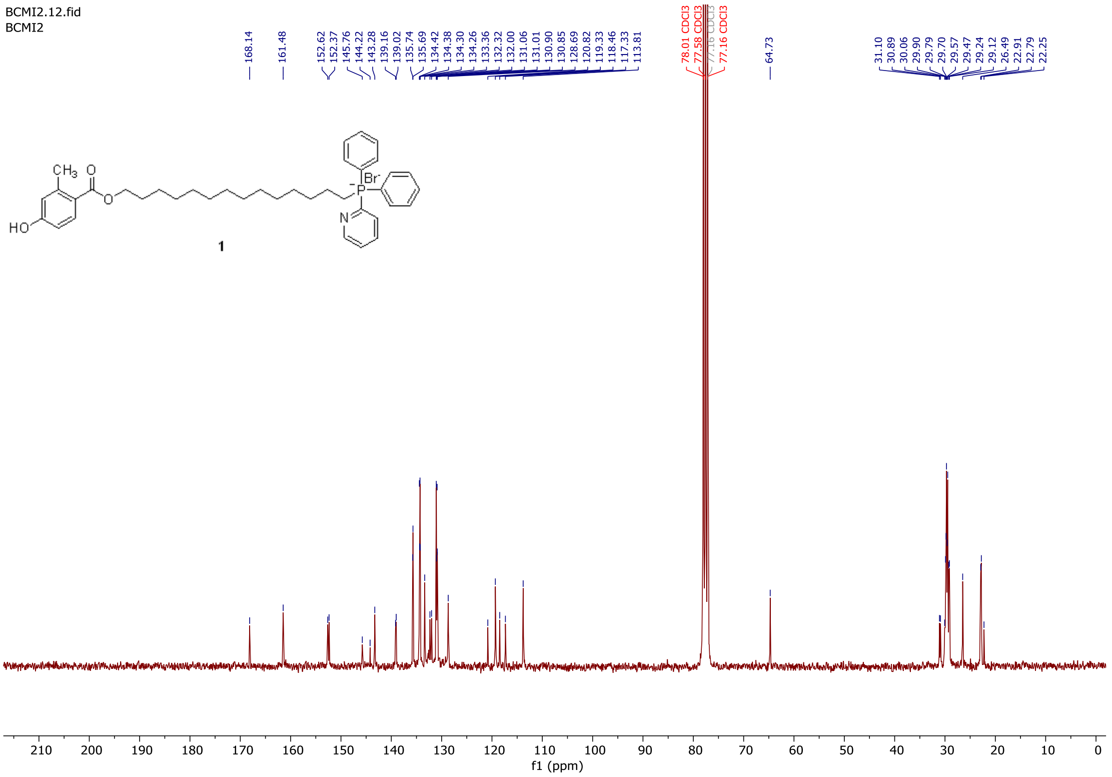

HPLC-MS

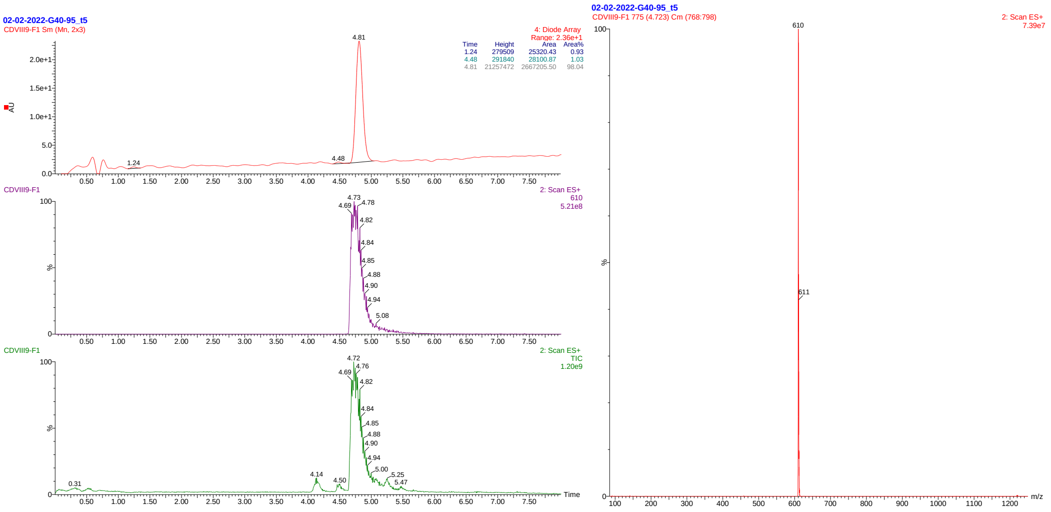

Compound **2**

^{1}H NMR ($CDCl_3$, 400 MHz)

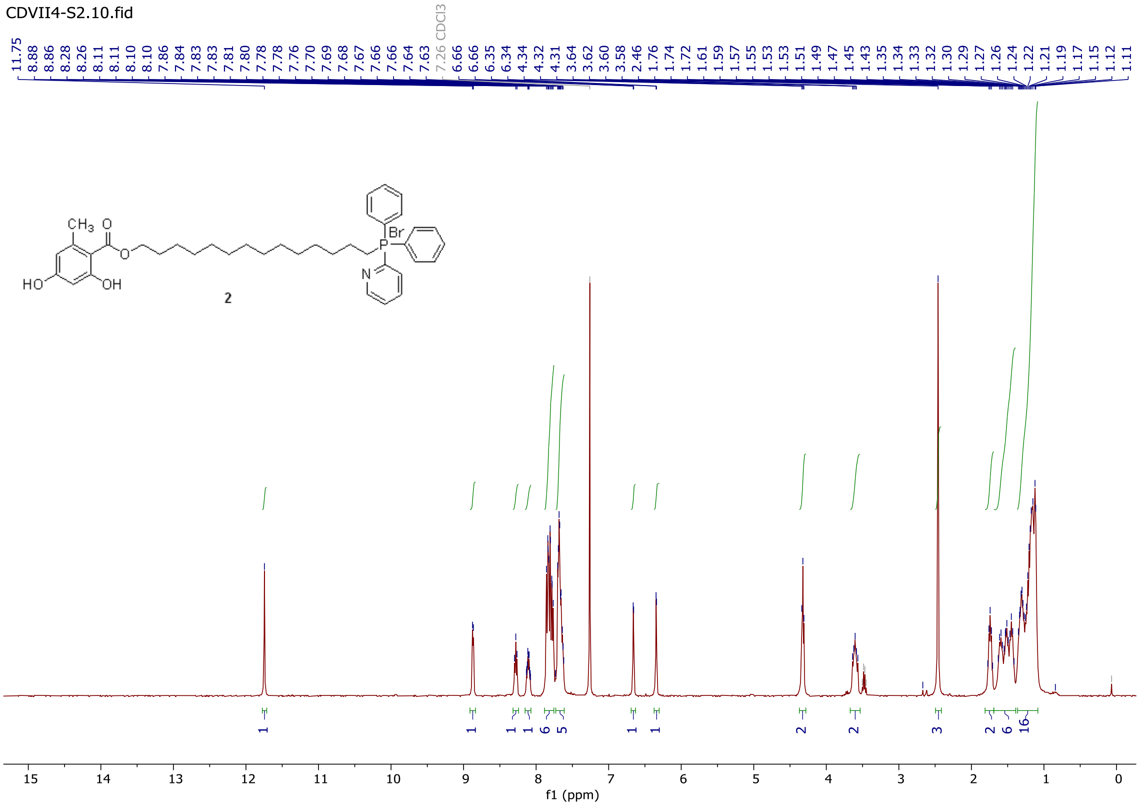

HPLC-MS

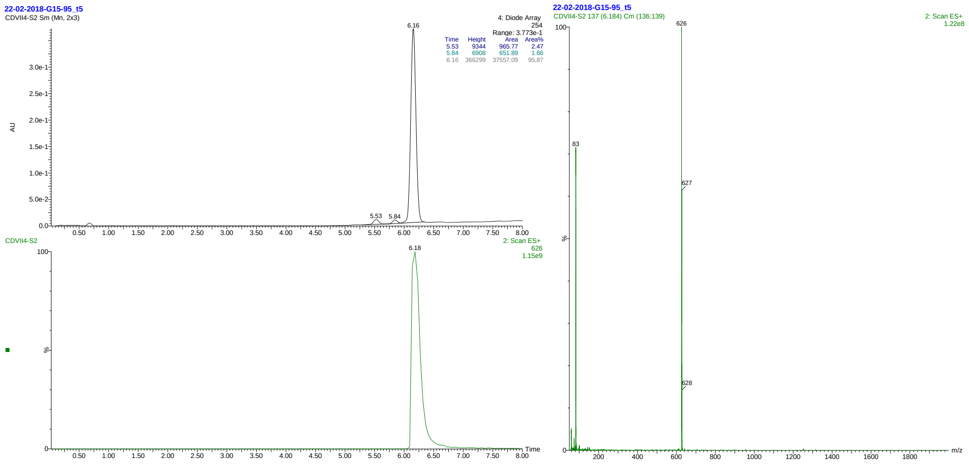

<u>Compound **7**</u>

^{1}H NMR ($CDCl_3$, 300 MHz)

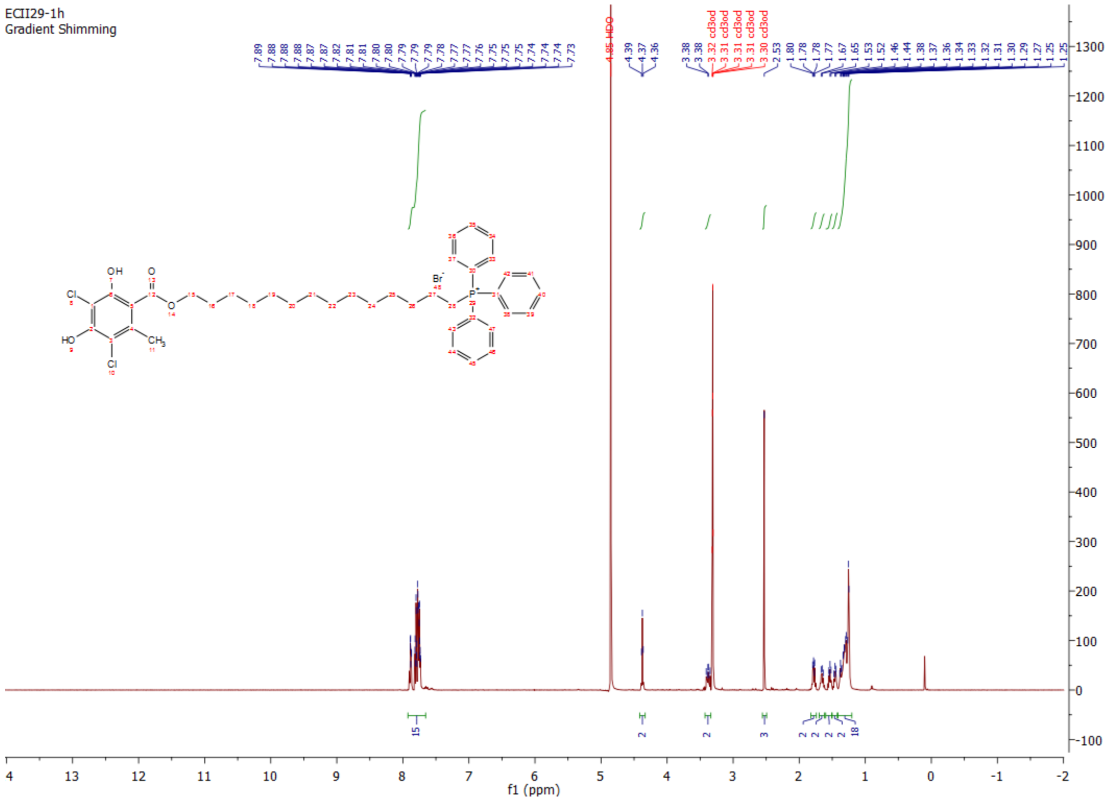

^{13}C NMR ($CDCl_3$, 75 MHz)

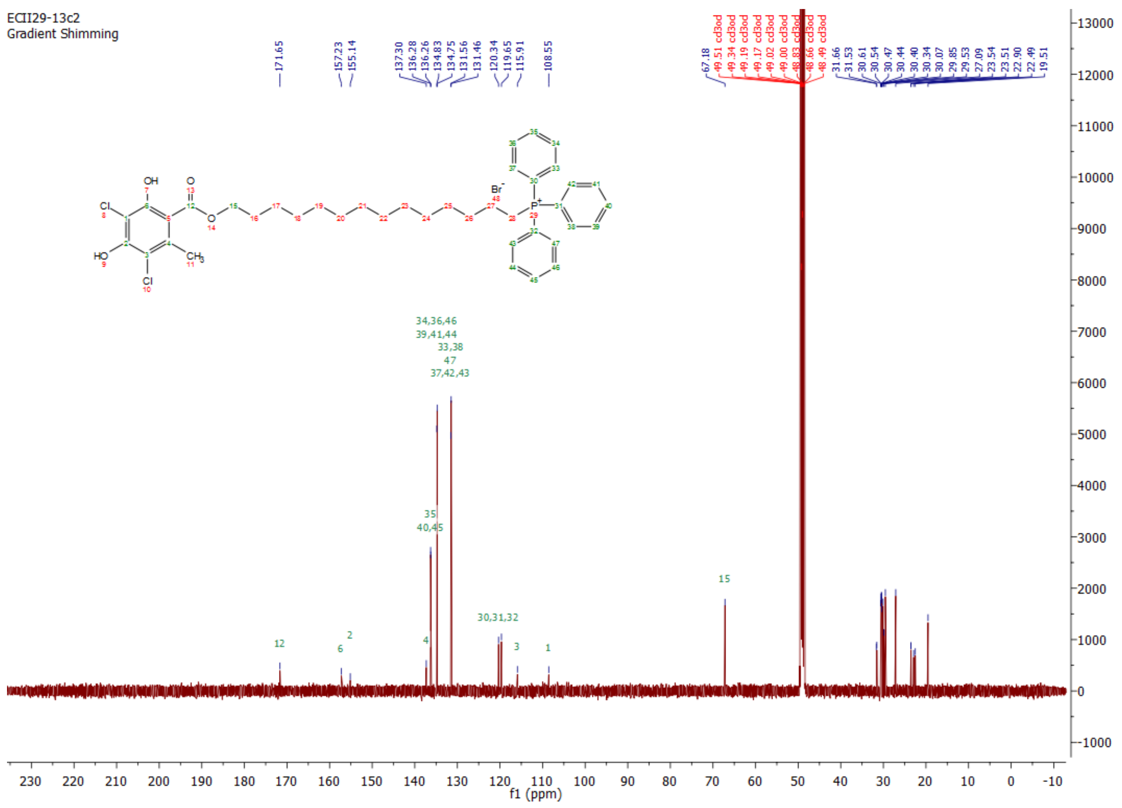

HPLC-MS

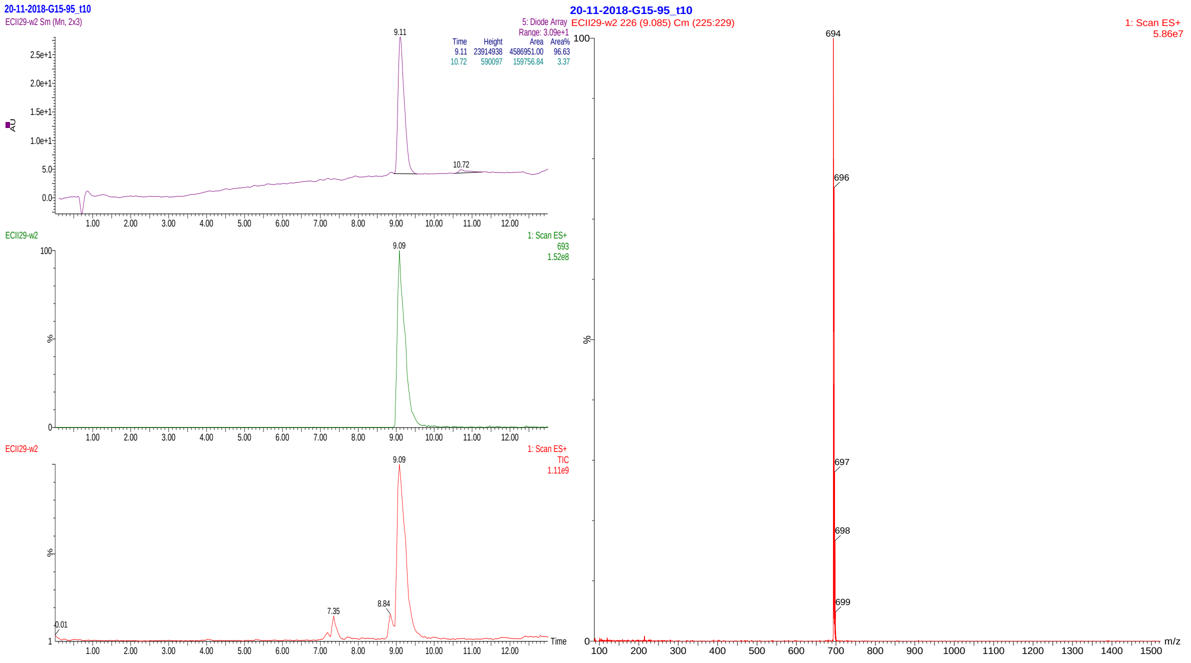

Supplement: Supplementary file 1 [file jm5c00631_si_001.pdf]
